# Supplementary material for: Scanning the Horizon for Environmental Applications of Genetically Modified Viruses Reveals Challenges for Their Environmental Risk Assessment
Source: Int J Mol Sci. 2024 Jan 25;25(3):1507. doi: 10.3390/ijms25031507 (PMC10855828; doi:10.3390/ijms25031507)
Supplement: Supplementary file 1 [file ijms-25-01507-s001.zip › Supplementary materials_IJMS.pdf]

Supplementary materials (Eckerstorfer et al. 2024)

Table S1: List of publications identified for GM viruses used as molecular tools in biotechnology applications, indicating their status of development and the use of GM viruses. VIGS: virus induced gene silencing; VAGE: virus aided gene expression; Status: assigned status of development; BR (L): Basic research (vector developed in laboratory conducting the study); R&D (SC): Research and development (vector shared in scientific community); NM/M (C): near market/market (vector available commercially)

No.	Virus used as vector	Purpose of the application	Purpose of the Study	Reference (study)	Status (vector)	Reference (vector)
1	Tobacco rattle virus (TRV); pTRV1&pTRV2	VIGS	Silencing genes involved in drought tolerance	[1]	R&D (SC)	[2]
2	Tobacco rattle virus (TRV); pTRV1&pTRV2	VIGS	Study of silenced and overexpressed gene involved in drought tolerance	[3]	R&D (SC)	[2]
3	-	Biology study (BPIV-3)	Development of molecular characterization to assess the role of homologous recombination events in the evolution of Bovine parainfluenza-3 virus	[4]	-	-
4	Nucleopolyhedrovirus (BmNPV)¸ BmNPV/DmTβH	VAGE	Baculovirus-mediated expression of protein in *Bombyx mori*	[5]	BR (L)	[6]
5	Tobacco rattle virus (TRV); pTRV1&pTRV2	VIGS	VIGS to suppress genes inducing programmed cell death to confer tolerance against abiotic stresses in plants.	[7]	R&D (SC)	[2,8,9]
6	Tobacco rattle virus (TRV); pTRV1&pTRV2	VIGS	VIGS of NbDODAL1 gene to modify the metabolism of aromatic compounds contributing to antimicrobial or antioxidative activity	[10]	R&D (SC)	[2]
7	Tobacco rattle virus (TRV); pTRV1&pTRV2	VIGS	VIGS to silence LOX2 expression to induce anti-herbivory responses against *S. littoralis* larvae	[11]	R&D (SC)	[2]

8	Nucleopolyhedrosis virus (BmNPV); pcDNA3.0, PGL3	VAGE	BmNPV-driven expression of miRNAs regulating gene expression in *Bombyx mori*	[12]	R&D (SC)	[13]
9	-	Virus-host specificity baculovirus	Study of virus-host specificity to aid the application of baculovirus in protein expression systems and the development of bio-control agents	[14]	-	-
10	Tobacco rattle virus (TRV); pTRV1&pTRV2	VIGS	VIGS of GhGPA expression in Arabidopsis increased susceptibility to Verticillium wilt	[15]	R&D (SC)	[2,16]
11	Tobacco rattle virus (TRV); pTRV1&pTRV2-LIC	VIGS	Development of TRV vectors for VIGS in *Centaurea spp*.	[17]	R&D (SC)	[18]
12	Baculovirus¸ pFastBac™ Dual Expression Vector	VAGE	Development of a baculovirus recombinant expression of transmembrane SVCV-G protein in insect cells, for use as whole-cell subunit vaccine through the oral and injection route in carp	[19]	NM/M (C)	Invitrogen / Thermo Fisher Scientific
13	Baculovirus¸ pFastBac™ Dual Expression Vector	VAGE	Baculovirus mediated expression of proteins for use in ELISA	[20]	NM/M (C)	Invitrogen / Thermo Fisher Scientific
14	Baculovirus; BmNPV K1	VAGE	Design of Baculovirus vectors for high efficiency protein expression	[21]	BR (L)	[22]
15	Cymbidium mosaic virus (CymMV); CymMV-Gateway	VIGS	CymMV-based VIGS system to be used with orchids	[23]	R&D (SC)	[24]
16	Tobacco rattle virus (TRV); pTRV1&pTRV2	VIGS	VIGS silencing of SlNAC61 transcription factor to elicit response to Tomato yellow leaf curl virus infection.	[25]	R&D (SC)	[2]

17	Hemorrhagic septicemia virus (VHSV); pVHSV-A-RFP	VAGE	GM VHSV to express IFN-proteins in flounder cells	[26]	R&D (SC)	[27]
18	Barley stripe mosaic virus (BSMV); BSMV-sg vectors	VAGE	Development of BSMV–based sgRNA delivery vector (BSMV-sg) in wheat	[28]	BR (L)	[29]
19	Tobacco rattle virus (TRV); pTRV1&pTRV2	VIGS	VIGS to elucidate the role of the PYL4 gene in response to drought in tomato	[30]	R&D (SC)	[9,31]
20	Cucumber green mottle mosaic virus (CGMMV); pXT- CGMMV	VAGE	CGMMV-based virus vectors to test use of subgenomic promoters	[32]	R&D (SC)	[33]
21	Barley stripe mosaic virus (BSMV); pCa-γbLIC	VIGS	VIGS to address cereal cyst nematode (CCN) infections in barley	[34]	R&D (SC)	[35]
22	Tobacco rattle virus (TRV); pTRV1&pTRV2	VIGS	VIGS-mediated knockdown of PAP3 led to the shriveling of pollen grains and male sterility in peppers	[36]	R&D (SC)	[2]
23	Tobacco rattle virus (TRV); pTRV1&pTRV2	VIGS	VIGS-mediated silencing of GhHD-1A and GhHD-1D in allotetraploid cotton significantly reduces the density of leaf hairs and affects the expression levels of other genes related to leaf trichome formation	[37]	R&D (SC)	[2]
24	Baculovirus¸ pFastBac™ Dual Expression Vector	VAGE	Baculovirus expression of Bovine parainfluenza virus type 3 (BPIV3) self-assembled GM-BPIV3 nanoparticles	[38]	NM/M (C)	Invitrogen / Thermo Fisher Scientific
25	Tobacco rattle virus (TRV); pTRV1&pTRV2	VIGS	VIGS to study the Ph-3-dependent resistance signaling pathway in tomato	[39]	R&D (SC)	[2,40]

26	Tobacco rattle virus (TRV); pTRV1&pTRV2	VIGS	TRV-based VIGS for gene function research in *Forsythia*	[41]	R&D (SC)	[2]
27	Barley stripe mosaic virus (BSMV); pSL038-1	VIGS	VIGS based functional study of TaNAC032 in Fusarium head blight in wheat	[42]	R&D (SC)	[43]
28	Tobacco rattle virus (TRV); pTRV1&pTRV2	VIGS	VIGS to downregulate SLB3 expression to reveal the function of the SLB3 gene under drought stress in tomato	[44]	R&D (SC)	[2]
29	Baculovirus; Bac-to-Bac™ Vector	VAGE	Development of pseudotyped recombinant baculovirus (Bacmid-GUS/VP28) for infection and expression in penaeid shrimps	[45]	NM/M (C)	Invitrogen / Thermo Fisher Scientific
30	Tobacco rattle virus (TRV); pTRV1&pTRV2	VIGS	TRV-based VIGS of a Phytoene Desaturase ortholog (LhPDS) in Lilium	[46]	R&D (SC)	[2]
31	Barley yellow striate mosaic virus (BYSMV); pBY-GR vector	VAGE	BYSMV a plant cytorhabdovirus as versatile expression platforms in small brown planthoppers and cereal plants.	[47]	R&D (SC)	[48]
32	Tobacco rattle virus (TRV); pTRV1&pTRV2	VIGS	Development of a TRV-based VIGS system in Chinese narcissus	[49]	R&D (SC)	[2]
33	Baculovirus¸ pFastBac™ Dual Expression Vector	VAGE	Development of a baculovirus insect cell system for expression of capsid protein of Infectious hypodermal and hematopoietic necrosis virus (IHHNV)	[50]	NM/M (C)	Invitrogen / Thermo Fisher Scientific

Table S2: List of publications identified for GM viruses for the development of vaccines for animals, indicating their status of development and the use of GM viruses Status: Status of development; BR: Basic research; R&D: Research and development; FT: Field trial

No.	Virus	Purpose of the application	Purpose of the Study	Reference (study)	Status	GMO
1	Canine distemper virus (CDV)	Dog vaccine	Evaluation of wild-type CDV and vaccine isolates to determine if the genetic differences among various strains result in significant antigenic differences	[51]	BR	No
2	Bovine herpesvirus 5	Cattle vaccine	A recombinant bovine herpesvirus 5 lacking thymidine kinase and glycoprotein E genes (BoHV-5gEΔTKΔ) was evaluated as a live experimental vaccine.	[52]	R&D	Yes
3	Feline Coronavirus (PBFIPV-DF-2, PBFIPV-DF-2-R3i)	Cat vaccine	Evaluation of a FcoV pair as possible vaccine candidates	[53]	R&D	Yes
4	Equine herpesvirus type-1 (EHV-1)	Horse vaccine	Assessment of intranasal and intramuscular vaccination of horses with a modified live equine herpesvirus type-1 (EHV-1) vaccine	[54]	R&D	No
5	Bovine rotavirus	Cattle vaccine	Test of adjuvanted vaccine containing recombinantly-expressed rotavirus virus-like particles	[55]	R&D	No
6	Betanodavirus	Fish vaccine	Review of antigenic preparations derived from the viral encephalopathy and retinopathy virus (betanodavirus) used in European sea bass	[56]	BR	No
7	Bovine herpesvirus type 5 (BoHV-5)	Cattle vaccine	Evaluation of inactivated, oil adjuvanted vaccine prepared with a GM BoHV-5 from which the genes coding for glycoprotein I (gI), glycoprotein E (gE) and membrane protein US9 were deleted (BoHV-5 gI/gE/US9−)	[57]	R&D	Yes
8	Pseudorabies virus (PRV)	Pig vector vaccine	GM PRV expressing siRNAs against the ORF7 of porcine reproductive and respiratory syndrome virus (HP-PRRSV)	[58]	BR	Yes
9	Baculovirus	Fish vector	Testing of GM baculoviruses expressing nine truncated membrane glycoproteins	[59]	R&D	Yes

		vaccine	of Cyprinid herpesvirus 2 (CyHV-2)			
10	Porcine reproductive and respiratory syndrome virus (PRRSV)	Pig vaccine	Evaluation of the cross-protection efficacy of two commercial PRRS modified-live virus (MLV) vaccines, derived from classical PRRSV (VR2332) and highly pathogenic (HP) PRRSV (TJM-F92), against a NADC30-like lineage in pigs	[60]	R&D	No
11	Porcine reproductive and respiratory syndrome virus (PRRSV)	Pig vaccine	Research into concerns about modified live vaccines (MLV)-derived PRRSV isolates	[61]	BR	No
12	Siniperca chuatsi rhabdovirus (SCRV)	Fish DNA vaccine	Determination of the antiviral immune response elicited by DNA vaccination in mandarin fish.	[62]	BR	No
13	Porcine reproductive and respiratory syndrome virus (PRRSV)	Pig vaccine	Safety and efficacy tests under experimental and field conditions for evaluation of K418DM1.1, a plaque-purified K418DM PRRSV vaccine	[63]	FT	Yes
14	Viral hemorrhagic septicemia virus (VHSV)	Fish vaccine (viral replicon particles)	Viral replicon particles / single-cycle viruses defective for function(s) needed for viral replication for safer vaccination of animals (olive flounders) against compared to attenuated live viruses	[64]	R&D	Yes
15	Betanodaviruses (Nodaviridae)	Fish vaccines	Assessment of viral encephalopathy and retinopathy (VER) disease agents: Striped Jack nervous necrosis virus (SJNNV), Tiger puffer nervous necrosis virus (TPNNV), Barfin flounder nervous necrosis virus (BFNNV), Red-spotted grouper nervous necrosis virus (RGNNV), turbot betanodavirus strain (TNV)	[65]	BR	No
16	Porcine reproductive and respiratory syndrome virus (PRRSV)	Pig vaccine	Assessment of a novel recombinant PRRSV, SD043, isolated from a pig farm experiencing disease in 2019	[66]	BR	No
17	Sindbis virus (SV)	Cattle SV-DNA prime-protein	Development of a SV replicase-based DNA vaccine against foot-and-mouth disease	[67]	BR	Yes

		boost vaccine				
18	Infectious spleen and kidney necrosis virus (ISKNV)	Fish vaccine	Assessment of a formalin-killed cell-cultured ISKNV vaccine mandarin fish in China as basis for GM ISKNV vaccines	[68]	BR	No
19	Newcastle disease virus (NDV)	Poultry / Chicken vaccine	Assessment of different NDV vaccines for efficacy against different isolates based on different vaccination regimes	[69]	BR	Yes
20	Porcine reproductive and respiratory syndrome virus (PRRSV)	Pig vaccine	Assessment of co-infection of a PRRSV type 1 modified live vaccine-like strain and a type 2 porcine circovirus (PCV2)	[70]	BR	No
21	Avian infectious bronchitis virus (IB)	Poultry vaccine	Non-GM IB vaccine variant testing	[71]	FT	No
22	Lumpy Skin Disease virus (LSDV) and Bluetongue virus (BTV)	Cattle vaccine	Developed of an inactivated, oil adjuvanted bivalent vaccine against both Lumpy Skin and Bluetongue disease based on LSDV Neethling strain and BTV4	[72]	BR	No
23	Infectious bursal disease virus (IBDV)	Poultry vaccine	Development and testing of a reassortment virus strain rGtVarVP2 effective against the novel variant IBDV	[73]	FT	Yes
24	Newcastle disease virus (NDV)	Poultry vaccine	Development and testing of an in-ovo NDV vaccine strain TS09-C to induce protective cellular immunity in chicks in the presence of maternal antibodies	[74]	FT	No
25	Classical Swine Fever Virus (CSFV)	Pig vaccine	Characterisation of genetic modifications in attenuated vaccine strains of CSFV	[75]	BR	No
26	African swine fever virus (ASFV)	Pig vaccine	Molecular evolution of circulating ASFV isolates and the important role of this gene in the evasion of the host immune system	[76]	BR	No
27	Pseudorabies virus (PrV)	Pig vector vaccine	Review of GM PrV as a powerful vector system for expression of foreign genes to	[77]	BR	Yes

			vaccinate against several infectious diseases of swine			
28	Semliki Forest virus (SFV)	Poultry DNA vaccine	A suicidal DNA vaccine based on a Semliki Forest virus (SFV) replicon was evaluated for the development of a vaccine against duck hepatitis virus type 1 (DHV-1)	[78]	R&D	Yes
29	Porcine reproductive and respiratory syndrome virus (PRRSV)	Pig vaccine	Testing of a recombinant PRRS virus (PRRSV) vectored vaccine candidate rPRRSV-E2, which expresses CSF virus (CSFV) E2 protein,	[79]	FT	Yes
30	Infectious bursal disease virus (IBDV)	Poultry DNA vaccine	Development of a DNA prime–protein boost vaccine against IBDV	[80]	R&D	Yes
31	Foot-and-mouth disease virus (FMDV)	Cattle vaccine	Characterisation of the effects of genetic changes in attenuated FMDV isolates	[81]	BR	Yes
32	Newcastle diseases virus (NDV) and Marek's disease virus (MDV)	Poultry vector vaccine	New generation vector vaccines against infectious laryngotracheitis in poultry stocks	[82]	R&D	Yes
33	Canarypox virus (CV)	Dog vector vaccine	Development of a recombinant canarypox virus "ALVAC-CDV-M-F-H/C5−" against Canine distemper virus (CDV) that contained CDV virus-like particles (VLPs) by using the CRISPR/Cas9 gene editing	[83]	R&D	Yes
34	Infectious hematopoietic necrosis virus (IHNV) and infectious pancreatic necrosis virus (IPNV)	Fish vaccine	Development of rIHNV-IPNV VP2 and rIHNV-IPNV VP2COE candidate vaccine against IHNV and IPNV in rainbow trout	[84]	R&D	Yes
35	Oxyeleotris marmoratus iridovirus (OMIV) and Oxyeleotris marmoratus	Fish vaccine	Development of a bivalent inactivated vaccine against OMIV and OMRV and evaluation of its protective effect in marbled sleepy goby *Oxyeleotris marmoratus*	[85]	R&D	No

	rhabdovirus (OMRV)					
36	Tembusu virus (TMUV)	Poultry vaccine	Development of a polytope DNA vaccine (pVAX1-rTEM) consisting of B-cell and T cell epitopes from the TMUV envelope (E) protein	[86]	R&D	Yes
37	Porcine circovirus 2 (PCV2)	-	Characterisation of genotypes and the evolution of Porcine circoviruses	[87]	BR	No
38	Koi herpesvirus (KHV) or Cyprinid Herpesvirus 3 (CyHV-3)	Fish vaccine	Development of a coupled plasmid expression vector for ORF149 and single walled carbon nanotubes (SWCNTs) for an anti-KHV vaccine	[88]	R&D	Yes
39	Porcine epidemic diarrhea virus (PEDV)	Pig vaccine	Assessment of the safety and efficacy of an attenuated live vaccine based on highly virulent genotype 2b porcine epidemic diarrhea virus in nursing piglets	[89]	R&D	No
40	Newcastle disease virus (NDV)	Poultry vaccine	Development of G7M mutated NDV as a promising genotype VII candidate vaccine	[90]	R&D	Yes
41	Nervous necrosis virus (NNV)	Fish vaccine	Investigation of humoral and cellular immunity responses of bath and oral vaccinations with BEI-inactivated NNV	[91]	BR	No
42	H5 highly pathogenic avian influenza virus (HPAIV)	-	Evaluation of different vaccine technologies for efficacy: 1) inactivated reverse genetic laboratory-generated virus encoding a clade 2.3.4.4 H5 hemagglutinin (HA) gene (rgH5), 2) recombinant turkey herpesvirus encoding a clade 2.2. H5 HA (rHVT-AI), and 3) recombinant replication-deficient alphavirus RNA particle vaccine encoding a clade 2.3.4.4 H5 HA (RP-H5)	[92]	BR	Yes
43	Porcine reproductive and respiratory syndrome virus (PRRSV)	Pig vaccine	Experimental testing of an experimental inactivated PRRSV vaccine	[93]	R&D	No
44	Viral hemorrhagic septicemia virus (VHSV)	Fish vaccine	Assessment of effectivity of differently treated VHSV vaccines in olive flounder	[94]	BR	No

45	Viral hemorrhagic septicemia virus (VHSV)	Fish vaccine	Development of a GM VHSV vaccine against virulent VHSV in olive flounder	[95]	R&D	Yes
46	Viral hemorrhagic septicemia virus (VHSV)	Fish vaccine	Development of a further developed GM VHSV vaccine in olive flounder	[96]	R&D	Yes
47	Viral hemorrhagic septicemia virus (VHSV)	Fish vaccine (viral replicon particles)	Development of a viral replicon particles rVHSV-GΔTM variant which inhibits the replication of wild-type VHSV when co-infected	[97]	R&D	Yes
48	Viral hemorrhagic septicemia virus (VHSV)	-	Review of VHSV mutants-based vaccines, particularly on the enhancement of immunogenicity, vaccine administration routes, safety to environments to support practical use in aquaculture farms	[98]	BR	-
49	Viral hemorrhagic septicemia virus (VHSV)	Fish vaccine (viral replicon particles)	Development rVHSV-ΔG a single cycle virus whose progenies are not infectious as a prophylactic vaccine	[99]	R&D	Yes
50	Orange-spotted grouper nervous necrosis virus (OGNNV) - Betanodavirus	Fish vaccine / Virus like particles	Demonstration that vaccination with OGNNV VLP induces humoral immune responses in orange-spotted grouper, *Epinephelus coioides*	[100]	R&D	No
51	Reticuloendotheliosis virus (REV)	Poultry vaccine	Demonstration of potential value of DNA vaccination in the prevention of REV infection and of codon optimization and GM DNA vaccines	[101]	BR	Yes
52	Tembusu virus (TMUV) Baculovirus - pFastBacDual vector	Poultry vaccine / virus-like particles (VLPs)	Demonstration that chimeric VLPs E protein of the DTMUV and HA2 protein of the H3N2 avian influenza virus is a candidate vaccine to control DTMUV infections in young duck	[102]	R&D	Yes
53	Fowl adenovirus (FAdV)	-	Comprehensive review of FAdV-4, including its epidemiology, pathogenesis, diagnostic detection, and vaccine strategies	[103]	BR	-
54	Baculovirus	Pig vaccine / virus-like	Baculovirus (rAc-Cap-SS) porcine circovirus-like particles as a novel subunit candidate vaccine for preventing PMWS	[104]	R&D	Yes

		particles (VLPs)				
55	Baculovirus	Cattle subunit vaccine	Silkworm baculovirus produced protein vaccine against foot-and-mouth disease virus (FMDV) Asia I/HNK/CHA/05	[105]	R&D	Yes
56	Pseudorabies virus (PRV)	Pig vaccine	Assessment of deletion-variant, attenuated PRV strain	[106]	R&D	No
57	Infectious bronchitis virus (IBV)	Poultry vaccine	Development of a highly attenuated nephropathogenic K2 IBV vaccine, with increased levels of protection against IBV	[107]	R&D	No
58	Porcine epidemic diarrhea virus (PEDV)	Pig vaccine	Characterisation of attenuated PEDV variants	[108]	BR	No
59	Highly pathogenic porcine reproductive and respiratory syndrome virus (HP-PRRSV)	Pig vaccine	Characterisation of live modified HP-PRRSV vaccine strains to regain infectivity	[109]	BR	No
60	African swine fever virus (ASFV)	Pig vaccine	Characterisation of the infectivity of ASFV△MGF100-1R deletion strain	[110]	BR	Yes
61	Adenovirus	Pig vector vaccine	Assessment of protective potential of 2 adenovirus-vectored cocktails containing ASFV antigens	[111]	R&D	Yes
62	Adenovirus	Pig vector vaccine	Demonstration that rAd-F1-Hsp70 has potent mucosal adjuvanticity in pigs, and is a candidate ASFV mucosal vaccine	[112]	R&D	Yes
63	Pseudorabies virus (PRV)	Pig vaccine	Development of a PRV candidate strain in which the protein kinase UL13 gene was deleted was constructed with the CRISPR/Cas9 system based on the recombinant pseudorabies virus (PRV) ZJ01-ΔgI/gE/TK	[113]	R&D	Yes
64	Pseudorabies virus (PRV)	Pig vaccine	Demonstrate of oral immunization against PRV infection in wild boar with Bartha vaccine	[114]	BR	No

65	Baculovirus	Sea Lion virus-like particles (VLPs)	Development of VLP with epitopes of Steller sea lion viruses V810 and V1415	[115]	BR	Yes
66	Kenyan sheep pox virus (KSPV)	Sheep vaccine	Assessment of effectivity of Kenyan sheep pox virus strain vaccine (KS1 O-180) against natural lumpy skin disease (LSD) infection under field conditions	[116]	BR	No
67	Lentivirus-based gene delivery system	Pig subunit marker vaccine	Development of Subunit vaccine to decrease vertical transmission of classical swine fever virus	[117]	R&D	Yes
68	Bovine viral diarrhea virus (BVDV)	Cattle vaccine	Assessment of effects of multivalent modified-live virus vaccine containing noncytopathic BVDV administered off-label to pregnant cattle	[118]	BR	No
69	Turkey herpesvirus (HVT)	Poultry vector ND vaccine	Assessment of immunity provided by a recombinant ND vaccine using HVT virus as vector (rHVT-ND)	[119]	R&D	Yes
70	African swine fever virus (ASFV)	-	Study to identify of ASFV antigens that induce a protective immune response	[120]	BR	Yes
71	Classical Swine Fever virus (CSFV)	Pig vaccine	Assessment of E2-CD154 subunit candidate vaccine in pregnant pigs	[121]	R&D	No
72	Bovine viral diarrhea virus (BVDV)	Cattle vaccine	Comparison of immune responses of calves exposed to a single dose of Bovela® bovine viral diarrhea virus (BVDV) live double deleted vaccine or a field strain virus (FSV) of BVDV type 2 (strain 890)	[122]	BR	Yes
73	Infectious Bursal Disease Virus (IBDV)	Poultry subunit DNA vaccine	Examination of the protective efficacy of a subunit DNA vaccine against IBDV infection in chickens	[123]	R&D	Yes
74	Foot and mouth disease virus (FMDV)	Cattle vaccine	Report of the construction and characterization of an infectious cDNA clone of the Indian vaccine strain of FMDV serotype O, IND-R2/75.	[124]	BR	Yes
75	Infectious Bursal Disease	Poultry subunit	Assessment of a live IBDV vaccine strain, IBDV MB-1	[125]	BR	No

	Virus (IBDV)	DNA vaccine				
76	Marek´s disease virus (MDV)	Poultry vaccines	Review discussing the current status of MD vaccines and their use as vector vaccines to control important viral poultry diseases	[126]	BR	-
77	Bovine viral diarrhea virus (BVDV)	Cattle vaccine	Construction of a new chimeric pestivirus "CP7_E1E2alf_TLA", based on the infectious cDNA of BVDV strain CP7	[127]	R&D	Yes
78	African swine fever virus (ASFV)	-	Review focusing on the challenges surrounding ASF vaccine design and development and existing knowledge gaps	[128]	BR	-
79	Turkey Herpesvirus (HVT)	Poultry vaccine	Assessment of a recombinant HVT-IBD vector vaccine and an intermediate plus live IBDV vaccine	[129]	R&D	Yes
80	Newcastle disease virus (NDV)	Poultry vaccine	Development of a thermostable, attenuated vaccine candidate strain NDV/rHR09	[130]	R&D	Yes
81	African swine fever virus (ASFV)	-	Review of information on the pathogenesis of ASF virus strains, the immune response to infection and prospects for developing vaccines	[131]	BR	-
82	Highly pathogenic avian influenza virus (HPAIV)	Poultry vaccine	Assessment of an inactivated influenza vaccine prepared from a non-pathogenic H5N1 AIV, A/duck/Hokkaido/Vac-3/2007 (H5N1)	[132]	R&D	Yes
83	Foot-and-mouth disease virus (FMDV)	Cattle vaccine	Evaluation of newly developed FMDV vaccine strains against the emerging serotype FMDV Asia1	[133]	R&D	Yes
84	Indian Peste des Petits Ruminants virus (PPRV)	Cattle vaccine	Assessment of virological and antigenic characteristics of two Indian Peste des Petits Ruminants (PPR) vaccine viruses namely "PPRV-Sungri/96" and "PPRV-AR/87"	[134]	BR	No
85	Marek's disease virus (MDV)	Poultry vaccine	Assessment of a deletion variant of the GM MDV variant GX0101, a field strain of MDV with a naturally occurring insertion of the reticuloendotheliosis virus long terminal repeat (LTR) fragment	[135]	R&D	Yes

86	Marek's disease virus (MDV)	Poultry vaccine	Development and assessment of a double deletion mutant MDV lacking virus-encoded meq and lorf9 as candidate vaccine	[136]	R&D	Yes
87	Newcastle disease virus (NDV), Tembusu virus (DTMUV)	Poultry vaccine	Assessment of a candidate vaccine aGM/prM + E against NDV and DTMUV transmissions in waterfowl raising areas in China	[137]	R&D	Yes
88	Porcine reproductive and respiratory syndrome virus (PRRSV)	Pig vaccine	Assessment of the efficacy of a novel BEI-inactivated PRRSV candidate vaccine in pigs	[138]	R&D	No
89	Classical swine fever virus (CSFV)	Pig vaccine	Examination of the induction of a strong T cellular response to confer a solid protection upon DNA-E2 vaccination against CSFV.	[139]	R&D	Yes
90	Classical swine fever virus (CSFV)	Pig vaccine	Assessment of a tandem-repeat multiple-epitope recombinant vaccine can protect pigs from CSFV challenge	[140]	R&D	Yes
91	African swine fever virus (ASFV)	-	Review of the research progress on ASFV in recent years	[141]	BR	-
92	Rabies virus (RV)	(Wild) dog vaccine	Applications of RV vector LBNSE expressing foreign antigens have shown considerable promise as vaccines against viral and bacteria diseases, e.g. canine distemper virus (CDV)	[142]	R&D	Yes
93	African swine fever virus (ASFV)	-	Overview of the current knowledge in innate and adaptive immune responses elicited by ASFV infection and different kinds of vaccine candidates	[143]	BR	-
94	Duck Orthoreovirus	-	Examination of effects of duck reovirus (DRV/GX-Y7) on waterfowl	[144]	BR	No
95	Porcine epidemic diarrhea virus (PEDV)	Pig vaccine	Investigation of the characteristics of a field strain of PEDV (PEDV strain SH) to develop candidate vaccines	[145]	BR	No
96	Newcastle disease virus	Poultry DNA	Assessment of immunization of chickens with Salmonella with pYL58 a NDV	[146]	BR	Yes

	(NDV)	vaccine	based DNA vaccine construct			
97	Infectious laryngotracheitis virus (ILTV	Poultry vector vaccine	Study of potential utility of ILTV-ΔgC–F as a bivalent candidate vaccine against both infectious laryngotracheitis and Newcastle disease	[147]	BR	Yes
98	Porcine circovirus type 2 (PCV2)	Pig vaccine	Evaluation of the efficacy of a new vaccine combining inactivated PCV2 and Mycoplasma hyorhinis	[148]	R&D	No
99	Infectious salmon anemia virus (ISAV)	Fish vaccine	A SAV-based replicon encoding the ISAV hemagglutinin-esterase, pSAV/HE, is an efficacious vaccine against infectious salmon anemia (ISA)	[149]	R&D	Yes
100	Pseudorabies virus (PRV)	Pig vaccine	Construction of a herpesvirus based bivalent vaccine with high-level expression of the ORF2 gene encoding the major antigen protein Cap of porcine circovirus type 2 (PCV2) in PRV	[150]	R&D	Yes
101	Avian infectious bronchitis virus (AIBV)	-	Characterization of a novel recombinant AIBV derived from GI-1, GI-13, GI-28, and GI-19 strains	[151]	BR	No
102	Foot-and-mouth disease virus (FMDV)	Cattle DNA vaccine	Assessment of FMD DNA vaccine with IL-18 formulated with PLGA nanoparticles	[152]	R&D	Yes
103	Pseudorabies virus (PRV)	Pig vector vaccine	Evaluation of PRV-based vaccine against Porcine circovirus type 3 (PCV3)	[153]	R&D	Yes
104	Largemouth bass virus (LMBV)	Fish DNA vaccine	A DNA vaccine expressing LMBV Major capsid protein might represent a potential LMBV vaccine candidate	[154]	R&D	Yes
105	Foot-and-mouth disease virus (FMDV)	Cattle vaccine	Construction of a modified FMD vaccine strain, C3 Resende-R	[155]	R&D	Yes
106	Singapore grouper iridovirus (SGIV)	Fish vaccine	The DNA vaccine construct pcDNA3.1–19R could induce protective immunity in grouper, and is a potential vaccine candidate for controlling SGIV disease	[156]	R&D	Yes
107	Rabbit hemorrhagic	Rabbit DNA	Construction of a novel DNA vaccine (pcDNA-VP60) expressing the RHDV capsid	[157]	R&D	Yes

	disease virus (RHDV)	vaccine	protein (VP60)			
108	Bovine viral diarrhea virus (BVDV) type 2	Cattle vaccine	Development of BVDV-2ΔNpro mutant which represents a promising candidate for an efficacious future live vaccine	[158]	R&D	Yes
109	Tilapia lake virus (TiLV)	Fish DNA vaccine	Efficacy by TiLV VP20 with different prime-boost vaccination regimens was evaluated in tilapia (DNA prime-protein boost immunization strategy was better than a solo vaccination)	[159]	R&D	Yes
110	Porcine circovirus type-2 (PCV2)	Pig vaccine	Evaluation of an inactivated PCV2-vaccine covalently conjugated with chitosan oligosaccharide	[160]	R&D	No
111	Newcastle disease virus (NDV)	Poultry vaccine	Development of GM NDV variants (rClon30-chGM-CSF and rClon30-flic) conferring enhanced protection in chicks	[161]	R&D	Yes
112	Turbot reddish body iridovirus (TRBIV)	Fish nanoparticle-based DNA vaccine	Chitosan nanoparticles were promising carriers for plasmid DNA vaccine pDNA-CS-NPs through oral vaccination	[162]	R&D	Yes
113	Porcine reproductive and respiratory syndrome virus (PRRSV)	Pig vaccine	Study providing guidance for the choice and use of PRRSV modified live virus vaccines to control NADC30-like virus infection in the field	[163]	BR	No
114	Infectious bronchitis virus (IBV)	Poultry vaccine	IBV strain H120 was successfully rescued as infectious clone by reverse genetics and a mutated variant constructed for vaccine use	[164]	R&D	Yes
115	Grass carp reovirus	Fish DNA vaccine	Evaluation of single walled carbon nanotubes (SWCNTs) as carriers for DNA vaccines for intramuscular injection	[165]	R&D	Yes
116	Foot-and-mouth disease virus (FMDV)	Cattle vaccine	Construction of a FLAGG tagged virus as candidate FMD vaccine which can be differentiated in vaccinated animals	[166]	BR	Yes

Table S3: List of publications identified for GM viruses as agents against plant pathogens, indicating their status of development and the use of GM viruses
Status: Status of development; BR: Basic research; R&D: Research and development

**No.**	**Virus**	**Purpose of the application**	**Purpose of the Study**	**Reference (study)**	**Status**	**GMO**
1	Citrus tristeza virus (CTV)	GM citrus plant development	GM citrus with CTV resistance	[167]	R&D	No
2	Citrus tristeza virus (CTV)	GM CTV as vector	Development of GM CTV-based expression vectors for plants	[168]	R&D	Yes
3	-	-	Vector-borne *Liberibacter* disease in plants	[169]	- (Review)	No
4	Citrus tristeza virus (CTV)	-	Effects of CTV strains on plants	[170]	BR	No
5	Citrus tristeza virus (CTV)	GM CTV as vector	GM CTV infected plants as trap crops for *Liberibacter* insect vector *Diaphorina citri*	[171]	R&D	Yes
6	Citrus yellow vein clearing virus (CYVCV)	-	Detection and management of CYVCV in citrus plants	[172]	- (Review)	No
7	Citrus tristeza virus (CTV)	-	Assessment of CYVCV interactions with infected citrus plants	[173]	BR	No
8	Citrus yellow vein clearing virus (CYVCV)	-	Serological detection of CYVCV in citrus plants	[174]	BR	No
9	Citrus psorosis virus (CPsV)	-	Immunodiagnostics of CPsV isolates	[175]	BR	No

Table S4: List of publications identified for GM viruses for the use as biocontrol agents, indicating their status of development and the use of GM viruses Status: Status of development; BR: Basic research; R&D: Research and development; FT: Field trial

No.	Virus	Purpose of the application	Purpose of the Study	Reference (study)	Status	GMO
1	Spodoptera litura nucleopolyhedrovirus (SpltNPV)	Biocontrol of agricultural pests	Biocontrol of *Spodoptera litura* as a primary insect pest of cotton in Pakistan due to reductions in pesticide use in GM cotton, resistant to *Helicoverpa armigera*	[176]	R&D	No
2	Spodoptera frugiperda multiple nucleopolyhedrovirus (SfMNPV)	Biocontrol of agricultural pests	Basic research of infectivity of SfMNPV occlusion bodies for *Spodoptera frugiperda*	[177]	BR	Yes
3	Chrysodeixis chalcites nucleopolyhedrovirus (ChchNPV)	Biocontrol of agricultural pests	Assessment of ChchSNPV-TF1 as a biological insecticide for control of *C. chalcites* in banana crops	[178]	R&D	No
4	A. ipsilon multiple nucleopolyhedrovirus (AgipMNPV)	Biocontrol of agricultural pests	Assessment of susceptibility of a grass-feeding caterpillar to a baculovirus	[179]	BR	No
5	Cryphonectria hypovirus (CHV1)	Hypovirulence as remedy to fungal blight disease	Detection of CHV1 virus in corticolous mites that are vectors of hypovirulence in blighted chestnut Castanea sativa	[180]	BR	No
6	Spodoptera exigua multiple nucleopolyhedrovirus (SeMNPV)	Biocontrol of agricultural pests	Assessment of key pathogenicity and virulence traits of SeMNPV isolates according to their principal transmission strategy	[181]	BR	No
7	Phthorimaea operculella granulovirus (PhopGV)	Biocontrol of agricultural pests	Development of a bioinsecticide based on PhopGV isolates against the pest Guatemala potato tuber moth	[182]	R&D	No

8	Condylorrhiza vestigialis nucleopolyhedrovirus (CoveNPV)	Biocontrol of forest pests	Research aimed at the use of CoveNPV in integrated pest management programs in Poplar crop	[183]	BR	No
9	Culex flavivirus (CxFV)	Identification of mosquito-associated virus	Characterisation of CxFV, a novel insect-specific flavivirus	[184]	BR	No
10	Nucleopolyhedroviruses	Biocontrol of agricultural pests	Study of virus-host specificity to aid the application of baculovirus in protein expression systems and the development of biocontrol agents	[14]	BR	No
11	Junonia coenia densovirus (JcDV)	Biocontrol of agricultural pests	Research on the potential of JcDV as a biological control agent against pests such as S. frugiperda	[185]	BR	No
12	Anticarsia gemmatalis multiple nucleopolyhedrovirus (AgMNPV) i	Biocontrol of agricultural pests	Assessment of AgMNPV as a viral insecticide	[186]	BR	No
13	Listeriaphage P100	Biocontrol of food pathogens	Assessment of resistance development in *Listeria monocytogenes* against phage-based control agents	[187]	BR	No
14	Spodoptera frugiperda multiple nucleocapsid nucleopolyhedrovirus (SfMNPV)	Biocontrol of agricultural pest	Assessment of SfMNPV isolates as the basis for a biopesticide for the control of *S. frugiperda* in Mexico	[188]	FT	No
15	Baculoviruses	Biocontrol of agricultural pest	Study on regulation of the neuronal activity of host caterpillars by baculoviruses	[189]	BR	Yes
16	L. obfuscata multiple nucleopolyhedrovirus	Biocontrol of tree / forest pests	Assessment of application of LyobMNPV against the host populations of Indian gypsy moth on apple and willow	[190]	FT	No

	(LyobMNPV)					
17	Baculovirus	Biocontrol of agricultural pests	Assessment of earthworm activity on the distribution and dynamics of baculovirus occlusion body populations in soil	[191]	BR	No
18	H. armigera single nucleopolyhedrovirus (HaSNPV)	Biocontrol of agricultural pests	Research into virus-mediated manipulation of host physiology	[192]	BR	Yes
19	Salivary gland hytrosaviruses (SGHVs)	Biocontrol of agricultural pests	Assessment of different SGHVs as potential biocontrol agent for tsetse vector control or as agents that may jeopardize tsetse mass rearing	[193]	BR	No
20	Glossina pallidipes salivary gland hytrosaviruses virus (GpSGHV)	Protection of mass rearing facilities	Development of potential targets for novel and sustainable molecular-based antiviral strategies to control viral infections in tsetse colonies	[194]	R&D	No
21	Baculovirus	Biocontrol of agricultural pests	Detection of covert baculovirus infections in the eastern spruce budworm, *Choristoneura fumiferana*	[195]	BR	No
22	Spodoptera exigua multiple nucleopolyhedrovirus (SeMNPV)	Biocontrol of agricultural pests	Assessment of activity of SeMNPV for biocontrol in Amaranth (Amaranthus hypocondriacus L.) cultures	[196]	R&D	No
23	Muscavirus (MdSGHV)	Biocontrol / Sterilizing agent	Assessment of MdSGHV as a sterilizing agent to control house fly populations	[197]	BR	No
24	C. capitata noravirus (CcaNV).	Protection of mass rearing facilities	Covert infections by CcaNV may impair the fitness of sterile Mediterranean fruit flies produced by SIT program	[198]	R&D	No
25	Chilo iridescent virus (CIV)	Biocontrol of agricultural pests	Insertion of a toxin gene into CIV provides opportunities to control a wide range of pest insects, such as weevils, using an iridovirus	[199]	R&D	Yes

26	Iflavirus (EVV-1)	Biocontrol of agricultural pests	Development of virus-based strategies against phytoplasma disease transmitted by the leafhopper vector *Euscelidius variegatus*	[200]	BR	No
27	Salmonid birnavirus (IPNV)	Biocontrol / Hypoinfectivity	Use of non-pathogenic IPNV to elicit resistance to VHSV superinfection in aquacultured fish species	[201]	BR	No
28	Lymantria dispar multiple nucleopolyhedrovirus-BNP (LdMNPV-BNP)	Biocontrol of forest pest	Assessment of LdMNPV-BNP as an active component of biopesticides that can be used during forest integrated pest management	[202]	BR	No
29	-	Biocontrol / immunocontraception	Assessment of biological control strategies in vertebrate pest management in Australia in case studies of rabbits, cane toads and carp	[203]	Overview biocontrol vertebrate pests Australia	-
30	Spodoptera exigua multiple nucleopolyhedrovirus (SeMNPV)	Biocontrol of agricultural pests	Development of gene knockout variants as SeMNPV candidate agents with significantly increased OB pathogenicity	[204]	BR	Yes
31	Spodoptera litura nucleopolyhedrovirus (SpltNPV)	Biocontrol of agricultural pests	Isolation of faster killing isolates as good candidates for biological control of *S. litura* in Pakistan	[205]	BR	No
32	Spodoptera frugiperda multiple nucleopolyhedrovirus (SfMNPV-NIC)	Biocontrol of agricultural pests	Assessment of gene knockout variants of SfMNPV-NIC with modified oral transmission efficacy	[206]	BR	Yes
33	Nucleopolyhedrovirus NPV (AgMNPV-79 or SfMNPV-6nd)	Biocontrol of agricultural pests	Assessment of the advantages and disadvantages of the simultaneous applications of NPV and *M. rileyi* for the management of either *A. gemmatalis* or *S. frugiperda*	[207]	BR	No

34	Lymantria mathura nucleopolyhedrovirus (LymaNPV)	Biocontrol of forest pests	Study on the basis of the interaction between *L. mathura* and LymaNPV	[208]	BR	No
35	Entomopoxviruses (EPVs)	Biocontrol of agricultural pests	Analysis of potential candidate isolates to control tortricid pests	[209]	BR	No
36	Helicoverpa armigera single nucleopolyhedrovirus (HearNPV)	Biocontrol of agricultural pests	Study of HearNPV interactions with host immunity to aid agricultural pest control	[210]	BR	No
37	Bacteriophage LPST144	Biocontrol of food pathogens	Assessment of bacteriophage LPST144 and its endolysin as potential antibacterial agents for Salmonella control in food industry	[211]	BR	No
38	Autographa californica multiple nucleopolyhedrovirus (AcMNPV)	Biocontrol of agricultural pests	A serial passage experiment shows long-term maintenance of a recombinant in serial passage and suggests an ecosystem safety risk	[212]	BR	Yes

References

1. Ahammed, G.J.; Li, X.; Wan, H.; Zhou, G.; Cheng, Y. SlWRKY81 reduces drought tolerance by attenuating proline biosynthesis in tomato. *Scientia Horticulturae* **2020**, *270*, 109444, doi:10.1016/j.scienta.2020.109444.
2. Liu, Y.; Schiff, M.; Marathe, R.; Dinesh-Kumar, S.P. Tobacco Rar1, EDS1 and NPR1/NIM1 like genes are required for N-mediated resistance to tobacco mosaic virus. *Plant J.* **2002**, *30*, 415–429, doi:10.1046/j.1365-313x.2002.01297.x.
3. Ahammed, G.J.; Li, X.; Yang, Y.; Liu, C.; Zhou, G.; Wan, H.; Cheng, Y. Tomato WRKY81 acts as a negative regulator for drought tolerance by modulating guard cell H2O2–mediated stomatal closure. *Environmental and Experimental Botany* **2020**, *171*, 103960, doi:10.1016/j.envexpbot.2019.103960.
4. Alatorre-García, T.A.; Fonseca-Coronado, S.; González-Candelas, F. Homologous recombination as a mechanism of genetic changes in bovine parainfluenza-3 virus. *Veterinary Microbiology* **2021**, *261*, 109185, doi:10.1016/j.vetmic.2021.109185.
5. Ali, A.M.; Lee, J.M.; Yoshida, M.; Sakashita, K.; Torii, J.; Kusakabe, T.; Hirashima, A. Expression and characterization of a recombinant Drosophila tyramine-β-hydroxylase in silkworm infected with recombinant baculovirus. *Journal of Asia-Pacific Entomology* **2012**, *15*, 567–572, doi:10.1016/j.aspen.2012.06.004.
6. Motohashi, T.; Shimojima, T.; Fukagawa, T.; Maenaka, K.; Park, E.Y. Efficient large-scale protein production of larvae and pupae of silkworm by Bombyx mori nuclear polyhedrosis virus bacmid system. *Biochemical and Biophysical Research Communications* **2005**, *326*, 564–569, doi:10.1016/j.bbrc.2004.11.060.
7. Bahieldin, A.; Alqarni, D.A.; Atef, A.; Gadalla, N.O.; Al-matary, M.; Edris, S.; Al-Kordy, M.A.; Makki, R.M.; Al-Doss, A.A.; Sabir, J.S.; et al. Suppression of PCD-related genes affects salt tolerance in Arabidopsis. *Comptes Rendus Biologies* **2016**, *339*, 105–114, doi:10.1016/j.crvi.2016.02.004.
8. Burch-Smith, T.M.; Schiff, M.; Liu, Y.; Dinesh-Kumar, S.P. Efficient virus-induced gene silencing in Arabidopsis. *Plant Physiol.* **2006**, *142*, 21–27, doi:10.1104/pp.106.084624.
9. Velásquez, A.C.; Chakravarthy, S.; Martin, G.B. Virus-induced gene silencing (VIGS) in Nicotiana benthamiana and tomato. *J. Vis. Exp.* **2009**, doi:10.3791/1292.
10. Bahramnejad, B.; Erickson, L.R.; Goodwin, P.H. Induction of expression and increased susceptibility due to silencing a 4,5-DOPA dioxygenase extradiol-like gene of Nicotiana benthamiana in the interaction with the hemibiotrophic pathogens, Colletotrichum destructivum, Colletotrichum orbiculare or Pseudomonas syringae pv. tabaci. *Plant Science* **2010**, *178*, 147–157, doi:10.1016/j.plantsci.2009.10.007.
11. Bejai, S.; Fridborg, I.; Ekbom, B. Varied response of Spodoptera littoralis against Arabidopsis thaliana with metabolically engineered glucosinolate profiles. *Plant Physiology and Biochemistry* **2012**, *50*, 72–78, doi:10.1016/j.plaphy.2011.07.014.
12. Cao, X.; Huang, Y.; Xia, D.; Qiu, Z.; Shen, X.; Guo, X.; Zhao, Q. BmNPV-miR-415 up-regulates the expression of TOR2 via Bmo-miR-5738. *Saudi Journal of Biological Sciences* **2017**, *24*, 1614–1619, doi:10.1016/j.sjbs.2015.09.020.
13. Chen, A.; Xia, D.; Qiu, Z.; Gao, P.; Tang, S.; Shen, X.; Zhu, F.; Zhao, Q. Expression of a vitelline membrane protein, BmVMP23, is repressed by bmo-miR-1a-3p in silkworm, Bombyx mori. *FEBS Letters* **2013**, *587*, 970–975, doi:10.1016/j.febslet.2013.02.030.
14. Chen, Y.-W.; Wu, C.-P.; Wu, T.-C.; Wu, Y.-L. Analyses of the transcriptome of Bombyx mori cells infected with either BmNPV or AcMNPV. *Journal of Asia-Pacific Entomology*

2018, *21*, 37–45, doi:10.1016/j.aspen.2017.10.009.

15. CHEN, B.; ZHANG, Y.; Yang, J.; Zhang, M.; Ma, Q.; Wang, X.; Ma, Z. The G-protein α subunit GhGPA positively regulates Gossypium hirsutum resistance to Verticillium dahliae via induction of SA and JA signaling pathways and ROS accumulation. *The Crop Journal* **2021**, *9*, 823–833, doi:10.1016/j.cj.2020.09.008.
16. Gao, X.; Wheeler, T.; Li, Z.; Kenerley, C.M.; He, P.; Shan, L. Silencing GhNDR1 and GhMKK2 compromises cotton resistance to Verticillium wilt. *Plant J.* **2011**, *66*, 293–305, doi:10.1111/j.1365-313X.2011.04491.x.
17. Deng, C.; ZHANG, F.; Wang, J.; Li, Y.; Huang, H.; Dai, S. Tobacco Rattle Virus-induced Phytoene Desaturase (PDS) Silencing in Centaurea cyanus. *Horticultural Plant Journal* **2021**, *7*, 159–166, doi:10.1016/j.hpj.2020.08.002.
18. Dong, Y.; Burch-Smith, T.M.; Liu, Y.; Mamillapalli, P.; Dinesh-Kumar, S.P. A ligation-independent cloning tobacco rattle virus vector for high-throughput virus-induced gene silencing identifies roles for NbMADS4-1 and -2 in floral development. *Plant Physiol.* **2007**, *145*, 1161–1170, doi:10.1104/pp.107.107391.
19. Embregts, C.; Rigaudeau, D.; Tacchi, L.; Pijlman, G.P.; Kampers, L.; Veselý, T.; Pokorová, D.; Boudinot, P.; Wiegertjes, G.F.; Forlenza, M. Vaccination of carp against SVCV with an oral DNA vaccine or an insect cells-based subunit vaccine. *Fish & Shellfish Immunology* **2019**, *85*, 66–77, doi:10.1016/j.fsi.2018.03.028.
20. Gao, S.; Wang, N.; Yang, J.; Sun, J.; Wang, Y.; Xia, D.; Tian, J.; Zhao, Y.; Feng, Y.; Zhou, Y.; et al. Development and evaluation of indirect double-antibody sandwich ELISA for rapid detection of Salmonid Alphavirus using Baculoviridae expressed E1 Protein. *Aquaculture* **2021**, *544*, 737081, doi:10.1016/j.aquaculture.2021.737081.
21. Gwak, W.-S.; Kim, H.-S.; Bae, J.-S.; Kim, T.-H.; Bae, S.-M.; Woo, S.-D. Development of a novel enhanced baculovirus expression vector via promoter combination. *Journal of Asia-Pacific Entomology* **2020**, *23*, 909–914, doi:10.1016/j.aspen.2020.07.016.
22. Chikako Ono; Tomonori Nakatsukasa; Yasuyuki Nishijima; Shin-ichiro Asano; Ken Sahara; Hisanori Bando. Construction of the BmNPV T3 Bacmid System and Its Application to the Functional Analysis of BmNPV he65. *J. Insect Biotechnol. Sericol.* **2007**, *76*, 3_161-3_167, doi:10.11416/jibs.76.3_161.
23. Hsieh, M.-H.; Lu, H.-C.; Pan, Z.-J.; Yeh, H.-H.; Wang, S.-S.; Chen, W.-H.; Chen, H.-H. Optimizing virus-induced gene silencing efficiency with Cymbidium mosaic virus in Phalaenopsis flower. *Plant Science* **2013**, *201-202*, 25–41, doi:10.1016/j.plantsci.2012.11.003.
24. Lu, H.-C.; Chen, H.-H.; Tsai, W.-C.; Chen, W.-H.; Su, H.-J.; Chang, D.C.-N.; Yeh, H.-H. Strategies for functional validation of genes involved in reproductive stages of orchids. *Plant Physiol.* **2007**, *143*, 558–569, doi:10.1104/pp.106.092742.
25. Huang, Y.; Li, T.; Xu, Z.-S.; Wang, F.; Xiong, A.-S. Six NAC transcription factors involved in response to TYLCV infection in resistant and susceptible tomato cultivars. *Plant Physiology and Biochemistry* **2017**, *120*, 61–74, doi:10.1016/j.plaphy.2017.09.020.
26. Kwak, J.S.; Kim, M.S.; Kim, K.H. Generation of a recombinant viral hemorrhagic septicemia virus (VHSV) expressing olive flounder (Paralichthys olivaceus) interferon-γ and its effects on type I interferon response and virulence. *Fish & Shellfish Immunology* **2017**, *68*, 530–535, doi:10.1016/j.fsi.2017.07.052.
27. Kim, M.S.; Kim, D.S.; Kim, K.H. Generation and characterization of NV gene-knockout recombinant viral hemorrhagic septicemia virus (VHSV) genotype IVa. *Dis. Aquat. Organ.* **2011**, *97*, 25–35, doi:10.3354/dao02394.

28. Li, T.; Hu, J.; Sun, Y.; Li, B.; Zhang, D.; Li, W.; Liu, J.; Li, D.; Gao, C.; Zhang, Y.; et al. Highly efficient heritable genome editing in wheat using an RNA virus and bypassing tissue culture. *Molecular Plant* **2021**, *14*, 1787–1798, doi:10.1016/j.molp.2021.07.010.
29. Hu, J.; Li, S.; Li, Z.; Li, H.; Song, W.; Zhao, H.; Lai, J.; Xia, L.; Li, D.; Zhang, Y. A barley stripe mosaic virus-based guide RNA delivery system for targeted mutagenesis in wheat and maize. *Mol. Plant Pathol.* **2019**, *20*, 1463–1474, doi:10.1111/mpp.12849.
30. Li, Y.; Zhang, X.; Jiang, J.; Zhao, T.; Xu, X.; Yang, H.; Li, J. Virus-induced gene silencing of SlPYL4 decreases the drought tolerance of tomato. *Horticultural Plant Journal* **2022**, *8*, 361–368, doi:10.1016/j.hpj.2021.06.005.
31. Ellison, E.E.; Nagalakshmi, U.; Gamo, M.E.; Huang, P.; Dinesh-Kumar, S.; Voytas, D.F. Multiplexed heritable gene editing using RNA viruses and mobile single guide RNAs. *Nature Plants* **2020**, *6*, 620–624, doi:10.1038/s41477-020-0670-y.
32. LIU, M.; LIU, L.; WU, H.; KANG, B.; GU, Q. Mapping subgenomic promoter of coat protein gene of Cucumber green mottle mosaic virus. *Journal of Integrative Agriculture* **2020**, *19*, 153–163, doi:10.1016/S2095-3119(19)62647-X.
33. Liu, L.; PENG, B.; Zhang, Z.; Wu, Y.; Miras, M.; Aranda, M.A.; Gu, Q. Exploring Different Mutations at a Single Amino Acid Position of Cucumber green mottle mosaic virus Replicase to Attain Stable Symptom Attenuation. *Phytopathology®* **2017**, *107*, 1080–1086, doi:10.1094/PHYTO-03-17-0107-R.
34. LUO, S.; KONG, L.; Peng, H.; HUANG, W.; CUI, J.; Liu, J.; QIAO, F.; JIAN, H.; PENG, D. Golden Promise barley (Hordeum vulgare) is a suitable candidate model host for investigation interaction with Heterodera avenae. *Journal of Integrative Agriculture* **2017**, *16*, 1537–1546, doi:10.1016/S2095-3119(16)61595-2.
35. Yuan, C.; Li, C.; Yan, L.; Jackson, A.O.; Liu, Z.; Han, C.; Yu, J.; Li, D. A high throughput barley stripe mosaic virus vector for virus induced gene silencing in monocots and dicots. *PLoS One* **2011**, *6*, e26468, doi:10.1371/journal.pone.0026468.
36. Ma, N.; Liu, C.; Yang, W.; Shen, H. PAP3 Regulates Stamen but Not Petal Development in Capsicum annuum L. *Horticultural Plant Journal* **2016**, *2*, 91–96, doi:10.1016/j.hpj.2016.02.009.
37. NIU, E.; CAI, C.; BAO, J.; WU, S.; ZHAO, L.; GUO, W. Up-regulation of a homeodomain-leucine zipper gene HD-1 contributes to trichome initiation and development in cotton. *Journal of Integrative Agriculture* **2019**, *18*, 361–371, doi:10.1016/S2095-3119(18)61914-8.
38. Qu, Z.; Li, M.; An, R.; Dai, H.; Yu, Y.; Li, C.; Cao, C.; Meng, Y.; Wang, J.; Gao, M. Self-assembled BPIV3 nanoparticles can induce comprehensive immune responses and protection against BPIV3 challenge by inducing dendritic cell maturation in mice. *Veterinary Microbiology* **2022**, *268*, 109415, doi:10.1016/j.vetmic.2022.109415.
39. Shah, S.R.A.; Tao, L.; Haijuan, C.; Lei, L.; Zheng, Z.; Junming, L.; Du Yongchen. Age-related Resistance and the Defense Signaling Pathway of Ph-3 Gene Against Phytophthora infestans in Tomatoes. *Horticultural Plant Journal* **2015**, *1*, 70–76, doi:10.16420/j.issn.2095-9885.2015-0019.
40. Chen, Y.-Y.; Lin, Y.-M.; Chao, T.-C.; Wang, J.-F.; Liu, A.-C.; Ho, F.-I.; Cheng, C.-P. Virus-induced gene silencing reveals the involvement of ethylene-, salicylic acid- and mitogen-activated protein kinase-related defense pathways in the resistance of tomato to bacterial wilt. *Physiol. Plant.* **2009**, *136*, 324–335, doi:10.1111/j.1399-3054.2009.01226.x.
41. Shen, J.; Si, W.; Wu, Y.; Xu, Y.; Wang, J.; Cheng, T.; Zhang, Q.; Pan, H. Establishment and Verification of An Efficient Virus-induced Gene Silencing System in Forsythia.

Horticultural Plant Journal **2021**, *7*, 81–88, doi:10.1016/j.hpj.2020.09.001.

42. Soni, N.; Altartouri, B.; Hegde, N.; Duggavathi, R.; Nazarian-Firouzabadi, F.; Kushalappa, A.C. TaNAC032 transcription factor regulates lignin-biosynthetic genes to combat Fusarium head blight in wheat. *Plant Science* **2021**, *304*, 110820, doi:10.1016/j.plantsci.2021.110820.
43. Cakir, C.; Scofield, S. Evaluating the ability of the barley stripe mosaic virus-induced gene silencing system to simultaneously silence two wheat genes. *Cereal Research Communications* **2008**, *36*, 217–222, doi:10.1556/CRC.36.2008.Suppl.B.18.
44. WANG, Z.; BAO, Y.; PEI, T.; WU, T.; DU, X.; HE, M.; WANG, Y.; LIU, Q.; YANG, H.; JIANG, J.; et al. Silencing the SLB3 transcription factor gene decreases drought stress tolerance in tomato. *Journal of Integrative Agriculture* **2020**, *19*, 2699–2708, doi:10.1016/S2095-3119(20)63350-0.
45. Wu, M.; Hu, Q.; Zhou, Y.; Guo, H. Development of a VP28-pseudotyped baculovirus expression system for efficient gene transfer in penaeid shrimps. *Aquaculture* **2021**, *541*, 736741, doi:10.1016/j.aquaculture.2021.736741.
46. Xu, H.; Xu, L.; Yang, P.; Cao, Y.; Tang, Y.; He, G.; Yuan, S.; Lei, J.; Ming, J. Virus-induced Phytoene Desaturase (PDS) Gene Silencing Using Tobacco Rattle Virus in Lilium × formolongi. *Horticultural Plant Journal* **2019**, *5*, 31–38, doi:10.1016/j.hpj.2018.10.002.
47. Xu, W.-Y.; Fang, X.-D.; Cao, Q.; Gao, Q.; Gao, D.-M.; Qiao, J.-H.; Zang, Y.; Xie, L.; Ding, Z.-H.; Yang, Y.-Z.; et al. A cytorhabdovirus-based expression vector in Nilaparvata lugens, Laodelphax striatellus, and Sogatella furcifera. *Insect Biochemistry and Molecular Biology* **2022**, *140*, 103703, doi:10.1016/j.ibmb.2021.103703.
48. Gao, Q.; Xu, W.-Y.; Yan, T.; Fang, X.-D.; Cao, Q.; Zhang, Z.-J.; Ding, Z.-H.; WANG, Y.; Wang, X.-B. Rescue of a plant cytorhabdovirus as versatile expression platforms for planthopper and cereal genomic studies. *New Phytol.* **2019**, *223*, 2120–2133, doi:10.1111/nph.15889.
49. Zhou, P.; Peng, J.; Zeng, M.; Wu, L.; Fan, Y.; Zeng, L. Virus-induced gene silencing (VIGS) in Chinese narcissus and its use in functional analysis of NtMYB3. *Horticultural Plant Journal* **2021**, *7*, 565–572, doi:10.1016/j.hpj.2021.04.009.
50. Zhu, Y.-P.; Li, C.; Wan, X.-Y.; Yang, Q.; Xie, G.S.; Huang, J. Delivery of plasmid DNA to shrimp hemocytes by Infectious hypodermal and hematopoietic necrosis virus (IHHNV) nanoparticles expressed from a baculovirus insect cell system. *Journal of Invertebrate Pathology* **2019**, *166*, 107231, doi:10.1016/j.jip.2019.107231.
51. Anis, E.; Holford, A.L.; Galyon, G.D.; Wilkes, R.P. Antigenic analysis of genetic variants of Canine distemper virus. *Veterinary Microbiology* **2018**, *219*, 154–160, doi:10.1016/j.vetmic.2018.03.014.
52. Anziliero, D.; Santos, C.; Brum, M.; Weiblen, R.; Chowdhury, S.I.; Flores, E.F. A recombinant bovine herpesvirus 5 defective in thymidine kinase and glycoprotein E is immunogenic for calves and confers protection upon homologous challenge and BoHV-1 challenge. *Veterinary Microbiology* **2011**, *154*, 14–22, doi:10.1016/j.vetmic.2011.03.019.
53. Bálint, Á.; Farsang, A.; Szeredi, L.; Zádori, Z.; Belák, S. Recombinant feline coronaviruses as vaccine candidates confer protection in SPF but not in conventional cats. *Veterinary Microbiology* **2014**, *169*, 154–162, doi:10.1016/j.vetmic.2013.10.015.
54. Bannai, H.; Nemoto, M.; Tsujimura, K.; Yamanaka, T.; Kokado, H.; Kondo, T.; Matsumura, T. Comparison of protective efficacies between intranasal and intramuscular vaccination of horses with a modified live equine herpesvirus type-1 vaccine. *Veterinary Microbiology* **2018**, *222*, 18–24, doi:10.1016/j.vetmic.2018.06.015.

55. Bristol, L.S.; Duhamel, G.E.; Zinckgraf, J.W.; Crabb, J.H.; Nydam, D.V. Effect of passive antibodies derived from rotavirus-like particles on neonatal calf diarrhea caused by rotavirus in an oral challenge model. *Journal of Dairy Science* **2021**, *104*, 11922–11930, doi:10.3168/jds.2020-19834.
56. Buonocore, F.; Nuñez-Ortiz, N.; Picchietti, S.; Randelli, E.; Stocchi, V.; Guerra, L.; Toffan, A.; Pascoli, F.; Fausto, A.M.; Mazzini, M.; et al. Vaccination and immune responses of European sea bass (Dicentrarchus labrax L.) against betanodavirus. *Fish & Shellfish Immunology* **2019**, *85*, 78–84, doi:10.1016/j.fsi.2017.11.039.
57. Campos, F.S.; Dezen, D.; Antunes, D.A.; Santos, H.F.; Arantes, T.S.; Cenci, A.; Gomes, F.; Lima, F.; Brito, W.; Filho, H.; et al. Efficacy of an inactivated, recombinant bovine herpesvirus type 5 (BoHV-5) vaccine. *Veterinary Microbiology* **2011**, *148*, 18–26, doi:10.1016/j.vetmic.2010.08.004.
58. Cao, S.; Guo, Q.; Wang, Y. Inhibition of highly pathogenic porcine reproductive and respiratory syndrome virus replication by recombinant pseudorabies virus-mediated RNA interference in piglets. *Veterinary Microbiology* **2015**, *181*, 212–220, doi:10.1016/j.vetmic.2015.10.016.
59. Cao, Z.; Liu, S.; Nan, H.; Zhao, K.; Xu, X.; Wang, G.; Ji, H.; Chen, H. Immersion immunization with recombinant baculoviruses displaying cyprinid herpesvirus 2 membrane proteins induced protective immunity in gibel carp. *Fish & Shellfish Immunology* **2019**, *93*, 879–887, doi:10.1016/j.fsi.2019.08.036.
60. Chai, W.; Liu, Z.; Sun, Z.; Su, L.; Zhang, C.; Huang, L. Efficacy of two porcine reproductive and respiratory syndrome (PRRS) modified-live virus (MLV) vaccines against heterologous NADC30-like PRRS virus challenge. *Veterinary Microbiology* **2020**, *248*, 108805, doi:10.1016/j.vetmic.2020.108805.
61. Chen, N.; Li, X.; Xiao, Y.; Li, S.; Zhu, J. Characterization of four types of MLV-derived porcine reproductive and respiratory syndrome viruses isolated in unvaccinated pigs from 2016 to 2020. *Research in Veterinary Science* **2021**, *134*, 102–111, doi:10.1016/j.rvsc.2020.12.007.
62. Chen, Z.-Y.; Lei, X.-Y.; Zhang, Q.-Y. The antiviral defense mechanisms in mandarin fish induced by DNA vaccination against a rhabdovirus. *Veterinary Microbiology* **2012**, *157*, 264–275, doi:10.1016/j.vetmic.2011.12.025.
63. Choi, H.-Y.; Lee, S.-H.; Ahn, S.-H.; Choi, J.-C.; Jeong, J.-Y.; Lee, B.-J.; Kang, Y.-L.; Hwang, S.-S.; Lee, J.-K.; Lee, S.-W.; et al. A chimeric porcine reproductive and respiratory syndrome virus (PRRSV)-2 vaccine is safe under international guidelines and effective both in experimental and field conditions. *Research in Veterinary Science* **2021**, *135*, 143–152, doi:10.1016/j.rvsc.2021.01.012.
64. Choi, M.G.; Kim, M.S.; Choi, T.-J.; Kim, K.H. Effect of CXCL12-expressing viral hemorrhagic septicemia virus replicon particles on leukocytes migration and vaccine efficacy in olive flounder (Paralichthys olivaceus). *Fish & Shellfish Immunology* **2019**, *89*, 378–383, doi:10.1016/j.fsi.2019.04.018.
65. Costa, J.Z.; Thompson, K.D. Understanding the interaction between Betanodavirus and its host for the development of prophylactic measures for viral encephalopathy and retinopathy. *Fish & Shellfish Immunology* **2016**, *53*, 35–49, doi:10.1016/j.fsi.2016.03.033.
66. Cui, X.-Y.; Xia, D.-S.; Huang, X.-Y.; Tian, X.-X.; Wang, T.; YANG, Y.; Wang, G.; Wang, H.-W.; SUN, Y.; Xiao, Y.-H.; et al. Recombinant characteristics, pathogenicity, and viral shedding of a novel PRRSV variant derived from twice inter-lineage recombination. *Veterinary Microbiology* **2022**, *271*, 109476, doi:10.1016/j.vetmic.2022.109476.
67. Dar, P.A.; Suryanaryana, V.S.; Nagarajan, G.; Reddy, G.R.; Dechamma, H.J.; Kondabattula, G. DNA prime-protein boost strategy with replicase-based DNA vaccine against foot-and-mouth disease in bovine calves. *Veterinary Microbiology* **2013**, *163*, 62–70, doi:10.1016/j.vetmic.2012.12.017.

68. Dong, C.; Xiong, X.; Luo, Y.; Weng, S.; Wang, Q.; He, J. Efficacy of a formalin-killed cell vaccine against infectious spleen and kidney necrosis virus (ISKNV) and immunoproteomic analysis of its major immunogenic proteins. *Veterinary Microbiology* **2013**, *162*, 419–428, doi:10.1016/j.vetmic.2012.10.026.
69. Dortmans, J.C.; Peeters, B.P.; Koch, G. Newcastle disease virus outbreaks: Vaccine mismatch or inadequate application? *Veterinary Microbiology* **2012**, *160*, 17–22, doi:10.1016/j.vetmic.2012.05.003.
70. Eclercy, J.; Larcher, T.; Andraud, M.; Renson, P.; Bernard, C.; Bigault, L.; Ledevin, M.; Paboeuf, F.; Grasland, B.; Rose, N.; et al. PCV2 co-infection does not impact PRRSV MLV1 safety but enhances virulence of a PRRSV MLV1-like strain in infected SPF pigs. *Veterinary Microbiology* **2020**, *244*, 108656, doi:10.1016/j.vetmic.2020.108656.
71. Erfanmanesh, A.; Ghalyanchilangeroudi, A.; Nikaein, D.; Hosseini, H.; Mohajerfar, T. Evaluation of inactivated vaccine of the variant 2 (IS-1494 /GI-23) genotype of avian infectious bronchitis. *Comparative Immunology, Microbiology and Infectious Diseases* **2020**, *71*, 101497, doi:10.1016/j.cimid.2020.101497.
72. Es-sadeqy, Y.; Bamouh, Z.; Ennahli, A.; Safini, N.; El Mejdoub, S.; Omari Tadlaoui, K.; Gavrilov, B.; El Harrak, M. Development of an inactivated combined vaccine for protection of cattle against lumpy skin disease and bluetongue viruses. *Veterinary Microbiology* **2021**, *256*, 109046, doi:10.1016/j.vetmic.2021.109046.
73. Fan, L.; Wang, Y.; Jiang, N.; GAO, L.; Li, K.; Gao, Y.; Cui, H.; Pan, Q.; Liu, C.; Zhang, Y.; et al. A reassortment vaccine candidate of the novel variant infectious bursal disease virus. *Veterinary Microbiology* **2020**, *251*, 108905, doi:10.1016/j.vetmic.2020.108905.
74. Fan, S.; Wu, Y.; Wang, H.; Shang, Y.; Luo, Q.; Zhang, T.; Zhang, R.; Zhang, W.; Luo, L.; Shao, H.; et al. In-ovo Newcastle disease virus vaccine strain TS09-C protects commercial chickens against Newcastle disease in the presence of maternally derived antibodies. *Poultry Science* **2020**, *99*, 2438–2443, doi:10.1016/j.psj.2020.01.006.
75. FAN, Y.; ZHAO, Q.; ZHAO, Y.; ZOU, X.; ZHANG, Z.; WANG, Q.; NING, Y. Heterogeneity and Secondary Structure Analysis of 3′ Untranslated Region in Classical Swine Fever Viruses. *Agricultural Sciences in China* **2011**, *10*, 142–148, doi:10.1016/S1671-2927(11)60316-7.
76. Frączyk, M.; Woźniakowski, G.; Kowalczyk, A.; Bocian, Ł.; Kozak, E.; Niemczuk, K.; Pejsak, Z. Evolution of African swine fever virus genes related to evasion of host immune response. *Veterinary Microbiology* **2016**, *193*, 133–144, doi:10.1016/j.vetmic.2016.08.018.
77. Freuling, C.M.; Müller, T.F.; Mettenleiter, T.C. Vaccines against pseudorabies virus (PrV). *Veterinary Microbiology* **2017**, *206*, 3–9, doi:10.1016/j.vetmic.2016.11.019.
78. Fu, Y.; Chen, Z.; Li, C.; Liu, G. Protective immune responses in ducklings induced by a suicidal DNA vaccine of the VP1 gene of duck hepatitis virus type 1. *Veterinary Microbiology* **2012**, *160*, 314–318, doi:10.1016/j.vetmic.2012.06.022.
79. Gao, F.; Jiang, Y.; Li, G.; Li, L.; Zhang, Y.; Yu, L.; Zheng, H.; Tong, W.; Zhou, Y.; Liu, C.; et al. Evaluation of immune efficacy of recombinant PRRSV vectored vaccine rPRRSV-E2 in piglets with maternal derived antibodies. *Veterinary Microbiology* **2020**, *248*, 108833, doi:10.1016/j.vetmic.2020.108833.
80. Gao, H.; Li, K.; GAO, L.; Qi, X.; Gao, Y.; Qin, L.; Wang, Y.; Wang, X. DNA prime–protein boost vaccination enhances protective immunity against infectious bursal disease virus in chickens. *Veterinary Microbiology* **2013**, *164*, 9–17, doi:10.1016/j.vetmic.2013.01.027.
81. Gao, H.; Wang, J.; Zhao, G.; Zhu, M.; He, Y.; Xin, A. Substitution 3A protein of foot-and-mouth disease virus of attenuated ZB strain rescued the viral replication and infection in bovine cells. *Research in Veterinary Science* **2020**, *128*, 145–152, doi:10.1016/j.rvsc.2019.11.001.

82. García, M. Current and future vaccines and vaccination strategies against infectious laryngotracheitis (ILT) respiratory disease of poultry. *Veterinary Microbiology* **2017**, *206*, 157–162, doi:10.1016/j.vetmic.2016.12.023.
83. Gong, Y.; Chen, T.; Feng, N.; Meng, X.; Sun, W.; Wang, T.; Zhao, Y.; Yang, S.; Song, X.; Li, W.; et al. A highly efficient recombinant canarypox virus-based vaccine against canine distemper virus constructed using the CRISPR/Cas9 gene editing method. *Veterinary Microbiology* **2020**, *251*, 108920, doi:10.1016/j.vetmic.2020.108920.
84. Guo, M.; Shi, W.; Wang, Y.; Wang, Y.; Chen, Y.; Li, D.; Ren, X.; Hua, X.; Tang, L.; Li, Y.; et al. Recombinant infectious hematopoietic necrosis virus expressing infectious pancreatic necrosis virus VP2 protein induces immunity against both pathogens. *Fish & Shellfish Immunology* **2018**, *78*, 187–194, doi:10.1016/j.fsi.2018.04.047.
85. Guo, X.; Zhou, Y.; Fu, X.; Lin, Q.; Liu, L.; Liang, H.; Niu, Y.; Li, N. Transcriptomic profiles reveal that inactivated iridovirus and rhabdovirus bivalent vaccine elicits robust adaptive immune responses against lethal challenge in marbled sleepy goby. *Fish & Shellfish Immunology* **2020**, *98*, 429–437, doi:10.1016/j.fsi.2020.01.047.
86. Han, K.; Zhao, D.; Liu, Y.; Huang, X.; Yang, J.; Liu, Q.; An, F.; Li, Y. Design and evaluation of a polytope construct with multiple B and T epitopes against Tembusu virus infection in ducks. *Research in Veterinary Science* **2016**, *104*, 174–180, doi:10.1016/j.rvsc.2015.09.011.
87. He, W.; Zhao, J.; Xing, G.; Li, G.; Wang, R.; Wang, Z.; Zhang, C.; Franzo, G.; Su, S.; Zhou, J. Genetic analysis and evolutionary changes of Porcine circovirus 2. *Molecular Phylogenetics and Evolution* **2019**, *139*, 106520, doi:10.1016/j.ympev.2019.106520.
88. Hu, F.; Li, Y.; Wang, Q.; Wang, G.; Zhu, B.; Wang, Y.; Zeng, W.; Yin, J.; Liu, C.; Bergmann, S.M.; et al. Carbon nanotube-based DNA vaccine against koi herpesvirus given by intramuscular injection. *Fish & Shellfish Immunology* **2020**, *98*, 810–818, doi:10.1016/j.fsi.2019.11.035.
89. Jang, G.; Won, H.; Lee, D.-U.; Noh, Y.-H.; Lee, S.-C.; Choi, H.-W.; Yoon, I.-J.; Lee, Y.J.; Sang Yoo, H.; Lee, C. Assessment of the safety and efficacy of an attenuated live vaccine based on highly virulent genotype 2b porcine epidemic diarrhea virus in nursing piglets. *Veterinary Microbiology* **2019**, *231*, 120–128, doi:10.1016/j.vetmic.2019.03.009.
90. Ji, Y.; Liu, T.; Du, Y.; Cui, X.; Yu, Q.; Wang, Z.; Zhang, J.; Li, Y.; Zhu, Q. A novel genotype VII Newcastle disease virus vaccine candidate generated by mutation in the L and F genes confers improved protection in chickens. *Veterinary Microbiology* **2018**, *216*, 99–106, doi:10.1016/j.vetmic.2018.01.021.
91. Kai, Y.-H.; Wu, Y.-C.; Chi, S.-C. Immune gene expressions in grouper larvae (Epinephelus coioides) induced by bath and oral vaccinations with inactivated betanodavirus. *Fish & Shellfish Immunology* **2014**, *40*, 563–569, doi:10.1016/j.fsi.2014.08.005.
92. Kapczynski, D.R.; Sylte, M.J.; Killian, M.L.; Torchetti, M.K.; Chrzastek, K.; Suarez, D.L. Protection of commercial turkeys following inactivated or recombinant H5 vaccine application against the 2015U.S. H5N2 clade 2.3.4.4 highly pathogenic avian influenza virus. *Veterinary Immunology and Immunopathology* **2017**, *191*, 74–79, doi:10.1016/j.vetimm.2017.08.001.
93. Karniychuk, U.U.; Saha, D.; Vanhee, M.; Geldhof, M.; Cornillie, P.; Caij, A.B.; Regge, N. de; Nauwynck, H.J. Impact of a novel inactivated PRRS virus vaccine on virus replication and virus-induced pathology in fetal implantation sites and fetuses upon challenge. *Theriogenology* **2012**, *78*, 1527–1537, doi:10.1016/j.theriogenology.2012.06.015.
94. Kim, H.J.; Park, J.S.; Choi, M.C.; Kwon, S.R. Comparison of the efficacy of Poly(I:C) immunization with live vaccine and formalin-killed vaccine against viral hemorrhagic septicemia virus (VHSV) in olive flounder (Paralichthys olivaceus). *Fish & Shellfish Immunology* **2016**, *48*, 206–211, doi:10.1016/j.fsi.2015.11.035.

95. Kim, M.S.; Kim, D.S.; Kim, K.H. Oral immunization of olive flounder (Paralichthys olivaceus) with recombinant live viral hemorrhagic septicemia virus (VHSV) induces protection against VHSV infection. *Fish & Shellfish Immunology* **2011**, *31*, 212–216, doi:10.1016/j.fsi.2011.05.003.
96. Kim, M.S.; Kim, K.H. Generation of recombinant viral hemorrhagic septicemia viruses (VHSVs) harboring G gene of hirame rhabdovirus (HIRRV) and their ability to induce serum neutralization activity in olive flounder (Paralichthys olivaceus). *Aquaculture* **2012**, *330-333*, 37–41, doi:10.1016/j.aquaculture.2011.12.018.
97. Kim, M.S.; Kim, K.H. Generation of VHSV replicon particles carrying transmembrane and C-terminal cytoplasmic region-deleted G gene (rVHSV-GΔTM) and comparison of vaccine efficacy with G gene-deleted VHSV (rVHSV-ΔG). *Fish & Shellfish Immunology* **2019**, *88*, 231–236, doi:10.1016/j.fsi.2019.02.048.
98. Kim, M.S.; Kim, K.H. Genetically engineered viral hemorrhagic septicemia virus (VHSV) vaccines. *Fish & Shellfish Immunology* **2019**, *95*, 11–15, doi:10.1016/j.fsi.2019.10.031.
99. Kim, M.S.; Park, J.S.; Kim, K.H. Generation of G gene-deleted viral hemorrhagic septicemia virus (VHSV) and evaluation of its vaccine potential in olive flounder (Paralichthys olivaceus). *Fish & Shellfish Immunology* **2015**, *45*, 666–671, doi:10.1016/j.fsi.2015.05.031.
100. Lai, Y.-X.; Jin, B.-L.; Xu, Y.; Huang, L.; Huang, R.-Q.; Zhang, Y.; Kwang, J.; He, J.-G.; Xie, J.-F. Immune responses of orange-spotted grouper, Epinephelus coioides, against virus-like particles of betanodavirus produced in Escherichia coli. *Veterinary Immunology and Immunopathology* **2014**, *157*, 87–96, doi:10.1016/j.vetimm.2013.10.003.
101. Li, K.; GAO, L.; Gao, H.; Qi, X.; Gao, Y.; Qin, L.; Wang, Y.; Wang, X. Protection of chickens against reticuloendotheliosis virus infection by DNA vaccination. *Veterinary Microbiology* **2013**, *166*, 59–67, doi:10.1016/j.vetmic.2013.04.031.
102. Li, L.; Zhang, Y.; Dong, J.; Zhang, J.; Zhang, C.; Qin, J.; Sun, M.; Xu, Z. Development of chimeric virus-like particles containing the E glycoprotein of duck Tembusu virus. *Veterinary Microbiology* **2019**, *238*, 108425, doi:10.1016/j.vetmic.2019.108425.
103. Li, P.H.; Zheng, P.P.; Zhang, T.F.; Wen, G.Y.; Shao, H.B.; Luo, Q.P. Fowl adenovirus serotype 4: Epidemiology, pathogenesis, diagnostic detection, and vaccine strategies. *Poultry Science* **2017**, *96*, 2630–2640, doi:10.3382/ps/pex087.
104. Li, W.; Wang, X.; Bai, J.; Ma, T.; Li, Z.; Li, Y.; Jiang, P. Construction and immunogenicity of recombinant porcine circovirus-like particles displaying somatostatin. *Veterinary Microbiology* **2013**, *163*, 23–32, doi:10.1016/j.vetmic.2012.11.045.
105. Li, Z.; Yin, X.; Yi, Y.; Li, X.; Li, B.; Lan, X.; Zhang, Z.; Liu, J. FMD subunit vaccine produced using a silkworm–baculovirus expression system: Protective efficacy against two type Asia1 isolates in cattle. *Veterinary Microbiology* **2011**, *149*, 99–103, doi:10.1016/j.vetmic.2010.10.022.
106. Liang, C.; Tong, W.; Zheng, H.; Liu, F.; Wu, J.; Li, G.; Zhou, E.; Tong, G. A high-temperature passaging attenuated Pseudorabies vaccine protects piglets completely against emerging PRV variant. *Research in Veterinary Science* **2017**, *112*, 109–115, doi:10.1016/j.rvsc.2017.02.008.
107. Lim, T.-H.; Kim, M.-S.; Jang, J.-H.; Lee, D.-H.; Park, J.-K.; Youn, H.-N.; Lee, J.-B.; Park, S.-Y.; Choi, I.-S.; Song, C.-S. Live attenuated nephropathogenic infectious bronchitis virus vaccine provides broad cross protection against new variant strains. *Poultry Science* **2012**, *91*, 89–94, doi:10.3382/ps.2011-01739.
108. Lin, C.-M.; Hou, Y.; Marthaler, D.G.; Gao, X.; Liu, X.; Zheng, L.; Saif, L.J.; Wang, Q. Attenuation of an original US porcine epidemic diarrhea virus strain PC22A via serial cell culture passage. *Veterinary Microbiology* **2017**, *201*, 62–71, doi:10.1016/j.vetmic.2017.01.015.

109. Liu, P.; Bai, Y.; Jiang, X.; Zhou, L.; Yuan, S.; Yao, H.; Yang, H.; Sun, Z. High reversion potential of a cell-adapted vaccine candidate against highly pathogenic porcine reproductive and respiratory syndrome. *Veterinary Microbiology* **2018**, *227*, 133–142, doi:10.1016/j.vetmic.2018.10.004.
110. Liu, Y.; LI, Y.; Xie, Z.; Ao, Q.; Di, D.; Yu, W.; Lv, L.; Zhong, Q.; Song, Y.; Liao, X.; et al. Development and in vivo evaluation of MGF100-1R deletion mutant in an African swine fever virus Chinese strain. *Veterinary Microbiology* **2021**, *261*, 109208, doi:10.1016/j.vetmic.2021.109208.
111. Lokhandwala, S.; Petrovan, V.; Popescu, L.; Sangewar, N.; Elijah, C.; Stoian, A.; Olcha, M.; Ennen, L.; Bray, J.; Bishop, R.P.; et al. Adenovirus-vectored African Swine Fever Virus antigen cocktails are immunogenic but not protective against intranasal challenge with Georgia 2007/1 isolate. *Veterinary Microbiology* **2019**, *235*, 10–20, doi:10.1016/j.vetmic.2019.06.006.
112. Lu, H.; Zhou, X.; Wu, Z.; Zhang, X.; Zhu, L.; Guo, X.; Zhang, Q.; Zhu, S.; Zhu, H.; Sun, H. Comparison of the mucosal adjuvanticities of two Toll-like receptor ligands for recombinant adenovirus-delivered African swine fever virus fusion antigens. *Veterinary Immunology and Immunopathology* **2021**, *239*, 110307, doi:10.1016/j.vetimm.2021.110307.
113. Lv, L.; Liu, X.; Jiang, C.; Wang, X.; Cao, M.; Bai, J.; Jiang, P. Pathogenicity and immunogenicity of a gI/gE/TK/UL13-gene-deleted variant pseudorabies virus strain in swine. *Veterinary Microbiology* **2021**, *258*, 109104, doi:10.1016/j.vetmic.2021.109104.
114. Maresch, C.; Lange, E.; Teifke, J.P.; Fuchs, W.; Klupp, B.; Müller, T.; Mettenleiter, T.C.; Vahlenkamp, T.W. Oral immunization of wild boar and domestic pigs with attenuated live vaccine protects against Pseudorabies virus infection. *Veterinary Microbiology* **2012**, *161*, 20–25, doi:10.1016/j.vetmic.2012.07.002.
115. McClenahan, S.D.; Bok, K.; Sosnovtsev, S.V.; Neill, J.D.; Burek, K.A.; Beckmen, K.B.; Smith, A.W.; Green, K.Y.; Romero, C.H. Expression and self-assembly of virus-like particles from two genotypes of marine vesiviruses and development of an ELISA for the detection of antibodies. *Veterinary Microbiology* **2010**, *142*, 184–192, doi:10.1016/j.vetmic.2009.09.057.
116. Molla, W.; Frankena, K.; Gari, G.; Jong, M.C. de. Field study on the use of vaccination to control the occurrence of lumpy skin disease in Ethiopian cattle. *Preventive Veterinary Medicine* **2017**, *147*, 34–41, doi:10.1016/j.prevetmed.2017.08.019.
117. Muñoz-González, S.; Sordo, Y.; Pérez-Simó, M.; Suárez, M.; Canturri, A.; Rodriguez, M.P.; Frías-Lepoureau, M.T.; Domingo, M.; Estrada, M.P.; Ganges, L. Efficacy of E2 glycoprotein fused to porcine CD154 as a novel chimeric subunit vaccine to prevent classical swine fever virus vertical transmission in pregnant sows. *Veterinary Microbiology* **2017**, *205*, 110–116, doi:10.1016/j.vetmic.2017.05.003.
118. Palomares, R.A.; Marley, S.M.; Givens, M.D.; Gallardo, R.A.; Brock, K.V. Bovine viral diarrhea virus fetal persistent infection after immunization with a contaminated modified-live virus vaccine. *Theriogenology* **2013**, *79*, 1184–1195, doi:10.1016/j.theriogenology.2013.02.017.
119. Palya, V.; Tatár-Kis, T.; Mató, T.; Felföldi, B.; Kovács, E.; Gardin, Y. Onset and long-term duration of immunity provided by a single vaccination with a turkey herpesvirus vector ND vaccine in commercial layers. *Veterinary Immunology and Immunopathology* **2014**, *158*, 105–115, doi:10.1016/j.vetimm.2013.11.008.
120. Pérez-Núñez, D.; Sunwoo, S.-Y.; Sánchez, E.G.; Haley, N.; García-Belmonte, R.; Nogal, M.; Morozov, I.; Madden, D.; Gaudreault, N.N.; Mur, L.; et al. Evaluation of a viral DNA-protein immunization strategy against African swine fever in domestic pigs. *Veterinary Immunology and Immunopathology* **2019**, *208*, 34–43, doi:10.1016/j.vetimm.2018.11.018.

121. Pérez-Pérez, D.; Sordo-Puga, Y.; Rodríguez-Moltó, M.P.; Sardina, T.; Santana, E.; Montero, C.; Ancizar, J.; Cabrera, Y.; Tuero, Á.; Naranjo, P.; et al. E2-CD154 vaccine candidate is safe and immunogenic in pregnant sows, and the maternal derived neutralizing antibodies protect piglets from classical swine fever virus challenge. *Veterinary Microbiology* **2021**, *260*, 109153, doi:10.1016/j.vetmic.2021.109153.
122. Platt, R.; Kesl, L.; Guidarini, C.; Wang, C.; Roth, J.A. Comparison of humoral and T-cell-mediated immune responses to a single dose of Bovela® live double deleted BVDV vaccine or to a field BVDV strain. *Veterinary Immunology and Immunopathology* **2017**, *187*, 20–27, doi:10.1016/j.vetimm.2017.03.003.
123. Pradhan, S.N.; Prince, P.R.; Madhumathi, J.; Arunkumar, C.; Roy, P.; Narayanan, R.B.; Antony, U. DNA vaccination with VP2 gene fragment confers protection against Infectious Bursal Disease Virus in Chickens. *Veterinary Microbiology* **2014**, *171*, 13–22, doi:10.1016/j.vetmic.2014.02.019.
124. Rajasekhar, R.; Hosamani, M.; Basagoudanavar, S.H.; Sreenivasa, B.P.; Tamil Selvan, R.P.; Saravanan, P.; Venkataramanan, R. Rescue of infective virus from a genome-length cDNA clone of the FMDV serotype O (IND-R2/75) vaccine strain and its characterization. *Research in Veterinary Science* **2013**, *95*, 291–297, doi:10.1016/j.rvsc.2013.01.026.
125. Ray, S.M.; Ashash, U.; Muthukumar, S. A field study on the evaluation of day-of-hatch and in grow-out application of live infectious bursal disease virus vaccine in broiler chickens. *Poultry Science* **2021**, *100*, 101252, doi:10.1016/j.psj.2021.101252.
126. Reddy, S.M.; Izumiya, Y.; Lupiani, B. Marek's disease vaccines: Current status, and strategies for improvement and development of vector vaccines. *Veterinary Microbiology* **2017**, *206*, 113–120, doi:10.1016/j.vetmic.2016.11.024.
127. Reimann, I.; Depner, K.; Utke, K.; Leifer, I.; Lange, E.; Beer, M. Characterization of a new chimeric marker vaccine candidate with a mutated antigenic E2-epitope. *Veterinary Microbiology* **2010**, *142*, 45–50, doi:10.1016/j.vetmic.2009.09.042.
128. Rock, D.L. Challenges for African swine fever vaccine development—"… perhaps the end of the beginning.". *Veterinary Microbiology* **2017**, *206*, 52–58, doi:10.1016/j.vetmic.2016.10.003.
129. Roh, J.-H.; Kang, M.; Wei, B.; Yoon, R.-H.; Seo, H.-S.; Bahng, J.-Y.; Kwon, J.-T.; Cha, S.-Y.; Jang, H.-K. Efficacy of HVT-IBD vector vaccine compared to attenuated live vaccine using in-ovo vaccination against a Korean very virulent IBDV in commercial broiler chickens. *Poultry Science* **2016**, *95*, 1020–1024, doi:10.3382/ps/pew042.
130. Ruan, B.; Liu, Q.; Chen, Y.; Niu, X.; Wang, X.; Zhang, C.; Guo, M.; Zhang, X.; Cao, Y.; Wu, Y. Generation and evaluation of a vaccine candidate of attenuated and heat-resistant genotype VIII Newcastle disease virus. *Poultry Science* **2020**, *99*, 3437–3444, doi:10.1016/j.psj.2020.01.034.
131. Sánchez-Cordón, P.J.; Montoya, M.; Reis, A.L.; Dixon, L.K. African swine fever: A re-emerging viral disease threatening the global pig industry. *The Veterinary Journal* **2018**, *233*, 41–48, doi:10.1016/j.tvjl.2017.12.025.
132. Shichinohe, S.; Okamatsu, M.; Yamamoto, N.; Noda, Y.; Nomoto, Y.; Honda, T.; Takikawa, N.; Sakoda, Y.; Kida, H. Potency of an inactivated influenza vaccine prepared from a non-pathogenic H5N1 virus against a challenge with antigenically drifted highly pathogenic avian influenza viruses in chickens. *Veterinary Microbiology* **2013**, *164*, 39–45, doi:10.1016/j.vetmic.2013.01.041.
133. Shin, S.H.; Jo, H.; Ko, M.-K.; Choi, J.-H.; You, S.-H.; Jo, H.-E.; Lee, M.J.; Kim, S.-M.; Kim, B.; Park, J.-H. Antigenic properties of a novel vaccine strain for type Asia1 foot-and-

mouth disease in pigs. *Veterinary Microbiology* **2020**, *248*, 108802, doi:10.1016/j.vetmic.2020.108802.

134. Singh, R.P.; De, U.K.; Pandey, K.D. Virological and antigenic characterization of two Peste des Petits Ruminants (PPR) vaccine viruses of Indian origin. *Comparative Immunology, Microbiology and Infectious Diseases* **2010**, *33*, 343–353, doi:10.1016/j.cimid.2008.12.003.
135. Su, S.; Cui, N.; Li, J.; Sun, P.; Li, H.; Li, Y.; Cui, Z. Deletion of the BAC sequences from recombinant meq-null Marek's disease (MD) virus increases immunosuppression while maintaining protective efficacy against MD. *Poultry Science* **2016**, *95*, 1504–1512, doi:10.3382/ps/pew067.
136. Sun, A.; Luo, J.; Wan, B.; Du, Y.; Wang, X.; Weng, H.; Cao, X.; Zhang, T.; Chai, S.; Zhao, D.; et al. Lorf9 deletion significantly eliminated lymphoid organ atrophy induced by meq-deleted very virulent Marek's disease virus. *Veterinary Microbiology* **2019**, *235*, 164–169, doi:10.1016/j.vetmic.2019.06.020.
137. Sun, M.; Dong, J.; Li, L.; Lin, Q.; Sun, J.; Liu, Z.; Shen, H.; Zhang, J.; Ren, T.; Zhang, C. Recombinant Newcastle disease virus (NDV) expressing Duck Tembusu virus (DTMUV) pre-membrane and envelope proteins protects ducks against DTMUV and NDV challenge. *Veterinary Microbiology* **2018**, *218*, 60–69, doi:10.1016/j.vetmic.2018.03.027.
138. Tabynov, K.; Sansyzbay, A.; Tulemissova, Z.; Tabynov, K.; Dhakal, S.; Samoltyrova, A.; Renukaradhya, G.J.; Mambetaliyev, M. Inactivated porcine reproductive and respiratory syndrome virus vaccine adjuvanted with Montanide™ Gel 01 ST elicits virus-specific cross-protective inter-genotypic response in piglets. *Veterinary Microbiology* **2016**, *192*, 81–89, doi:10.1016/j.vetmic.2016.06.014.
139. Tarradas, J.; Argilaguet, J.M.; Rosell, R.; Nofrarías, M.; Crisci, E.; Córdoba, L.; Pérez-Martín, E.; Díaz, I.; Rodríguez, F.; Domingo, M.; et al. Interferon-gamma induction correlates with protection by DNA vaccine expressing E2 glycoprotein against classical swine fever virus infection in domestic pigs. *Veterinary Microbiology* **2010**, *142*, 51–58, doi:10.1016/j.vetmic.2009.09.043.
140. Tian, H.; Hou, X.; Wu, J.; Chen, Y.; Shang, Y.; Yin, S.; Zhang, K.; Liu, X. A promising multiple-epitope recombinant vaccine against classical swine fever virus. *Veterinary Immunology and Immunopathology* **2014**, *157*, 59–64, doi:10.1016/j.vetimm.2013.10.014.
141. Wang, F.; ZHANG, H.; Hou, L.; Yang, C.; Wen, Y. Advance of African swine fever virus in recent years. *Research in Veterinary Science* **2021**, *136*, 535–539, doi:10.1016/j.rvsc.2021.04.004.
142. Wang, F.-X.; Zhang, S.-Q.; Zhu, H.-W.; Yang, Y.; Sun, N.; Tan, B.; Li, Z.-G.; Cheng, S.-P.; Fu, Z.F.; Wen, Y.-J. Recombinant rabies virus expressing the H protein of canine distemper virus protects dogs from the lethal distemper challenge. *Veterinary Microbiology* **2014**, *174*, 362–371, doi:10.1016/j.vetmic.2014.10.023.
143. Wang, T.; Sun, Y.; Huang, S.; Qiu, H.-J. Multifaceted Immune Responses to African Swine Fever Virus: Implications for Vaccine Development. *Veterinary Microbiology* **2020**, *249*, 108832, doi:10.1016/j.vetmic.2020.108832.
144. Wang, W.; Liang, J.; Shi, M.; Chen, G.; Huang, Y.; ZHANG, Y.; Zhao, Z.; Wang, M.; Li, M.; Mo, M.; et al. The diagnosis and successful replication of a clinical case of Duck Spleen Necrosis Disease: An experimental co-infection of an emerging unique reovirus and Salmonella indiana reveals the roles of each of the pathogens. *Veterinary Microbiology* **2020**, *246*, 108723, doi:10.1016/j.vetmic.2020.108723.

145. Wang, X.-W.; Wang, M.; Zhan, J.; Liu, Q.-Y.; Fang, L.; Zhao, C.; Jiang, P.; Li, Y.-F.; Bai, J. Pathogenicity and immunogenicity of a new strain of porcine epidemic diarrhea virus containing a novel deletion in the N gene. *Veterinary Microbiology* **2020**, *240*, 108511, doi:10.1016/j.vetmic.2019.108511.

146. Wang, Z.; WANG, Y.; Sun, C.; Zhao, X.; Sun, M.; Gao, X.; Jia, F.; Zhang, T.; Ge, C.; Zhang, X.; et al. Protection against genotype VII Newcastle disease virus challenge by a minicircle DNA vaccine coexpressing F protein and chicken IL-18 adjuvant. *Veterinary Microbiology* **2022**, *270*, 109474, doi:10.1016/j.vetmic.2022.109474.

147. Wei, X.; Shao, Y.; Han, Z.; Sun, J.; Liu, S. Glycoprotein-C-gene-deleted recombinant infectious laryngotracheitis virus expressing a genotype VII Newcastle disease virus fusion protein protects against virulent infectious laryngotracheitis virus and Newcastle disease virus. *Veterinary Microbiology* **2020**, *250*, 108835, doi:10.1016/j.vetmic.2020.108835.

148. Wei, Y.-W.; Zhu, H.-Z.; Huang, L.-P.; Xia, D.-L.; Wu, H.-L.; Bian, H.-Q.; Feng, L.; Liu, C.-M. Efficacy in pigs of a new inactivated vaccine combining porcine circovirus type 2 and Mycoplasma hyorhinis. *Veterinary Microbiology* **2020**, *242*, 108588, doi:10.1016/j.vetmic.2020.108588.

149. Wolf, A.; Hodneland, K.; Frost, P.; Hoeijmakers, M.; Rimstad, E. Salmonid alphavirus-based replicon vaccine against infectious salmon anemia (ISA): Impact of immunization route and interactions of the replicon vector. *Fish & Shellfish Immunology* **2014**, *36*, 383–392, doi:10.1016/j.fsi.2013.12.018.

150. Wu, X.; Wu, H.; Wang, H.; Luo, L.; Wang, J.; Wu, B.; He, Q.; Cao, G.; Lei, Y.; Chen, X.; et al. A new strategy to develop pseudorabies virus-based bivalent vaccine with high immunogenicity of porcine circovirus type 2. *Veterinary Microbiology* **2021**, *255*, 109022, doi:10.1016/j.vetmic.2021.109022.

151. Yan, W.; Qiu, R.; Wang, F.; Fu, X.; Li, H.; Cui, P.; Zhai, Y.; Li, C.; Zhang, L.; Gu, K.; et al. Genetic and pathogenic characterization of a novel recombinant avian infectious bronchitis virus derived from GI-1, GI-13, GI-28, and GI-19 strains in Southwestern China. *Poultry Science* **2021**, *100*, 101210, doi:10.1016/j.psj.2021.101210.

152. Yang, Y.; Teng, Z.; Lu, Y.; Luo, X.; Mu, S.; Ru, J.; Zhao, X.; Guo, H.; Ran, X.; Wen, X.; et al. Enhanced immunogenicity of foot and mouth disease DNA vaccine delivered by PLGA nanoparticles combined with cytokine adjuvants. *Research in Veterinary Science* **2021**, *136*, 89–96, doi:10.1016/j.rvsc.2021.02.010.

153. Yao, L.; Cheng, Y.; Wu, H.; Ghonaim, A.H.; Fan, S.; Li, W.; He, Q. The construction and immunogenicity analyses of a recombinant pseudorabies virus with porcine circovirus type 3 capsid protein co-expression. *Veterinary Microbiology* **2022**, *264*, 109283, doi:10.1016/j.vetmic.2021.109283.

154. Yi, W.; Zhang, X.; Zeng, K.; Xie, D.; Song, C.; Tam, K.; Liu, Z.; Zhou, T.; LI, W. Construction of a DNA vaccine and its protective effect on largemouth bass (Micropterus salmoides) challenged with largemouth bass virus (LMBV). *Fish & Shellfish Immunology* **2020**, *106*, 103–109, doi:10.1016/j.fsi.2020.06.062.

155. You, S.-H.; Jo, H.-E.; Choi, J.-H.; Ko, M.-K.; Shin, S.H.; Lee, M.J.; Kim, S.-M.; Kim, B.; Park, J.-H. Evaluation of novel inactivated vaccine for type C foot-and-mouth disease in cattle and pigs. *Veterinary Microbiology* **2019**, *234*, 44–50, doi:10.1016/j.vetmic.2019.05.016.

156. Yu, N.-T.; Zheng, X.; Liu, Z.-X. Protective immunity induced by DNA vaccine encoding viral membrane protein against SGIV infection in grouper. *Fish & Shellfish Immunology* **2019**, *92*, 649–654, doi:10.1016/j.fsi.2019.06.051.

157. Yuan, D.; Qu, L.; Liu, J.; Guo, D.; Jiang, Q.; Lin, H.; Si, C. DNA vaccination with a gene encoding VP60 elicited protective immunity against rabbit hemorrhagic disease virus. *Veterinary Microbiology* **2013**, *164*, 1–8, doi:10.1016/j.vetmic.2013.01.021.

158. Zemke, J.; König, P.; Mischkale, K.; Reimann, I.; Beer, M. Novel BVDV-2 mutants as new candidates for modified-live vaccines. *Veterinary Microbiology* **2010**, *142*, 69–80, doi:10.1016/j.vetmic.2009.09.045.

159. Zeng, W.; Wang, Y.; Chen, X.; Wang, Q.; Bergmann, S.M.; Yang, Y.; Wang, Y.; LI, B.; Lv, Y.; Li, H.; et al. Potency and efficacy of VP20-based vaccine against tilapia lake virus using different prime-boost vaccination regimens in tilapia. *Aquaculture* **2021**, *539*, 736654, doi:10.1016/j.aquaculture.2021.736654.

160. Zhang, G.; Jia, P.; Cheng, G.; Jiao, S.; Ren, L.; Ji, S.; Hu, T.; Liu, H.; Du, Y. Enhanced immune response to inactivated porcine circovirus type 2 (PCV2) vaccine by conjugation of chitosan oligosaccharides. *Carbohydrate Polymers* **2017**, *166*, 64–72, doi:10.1016/j.carbpol.2017.02.058.

161. Zhang, T.; Liu, Y.; Wang, H.; Zhang, X.; Zhu, S.; Xu, P.; Yin, J.; Ren, G.; Liu, J.; Li, D. Recombinant NDV expressing cytokines or fliC confers a quick immune response against NDV challenge and resistance to maternal antibody. *Veterinary Microbiology* **2016**, *196*, 107–117, doi:10.1016/j.vetmic.2016.10.008.

162. Zheng, F.; Liu, H.; Sun, X.; Zhang, Y.; Zhang, B.; Teng, Z.; Hou, Y.; Wang, B. Development of oral DNA vaccine based on chitosan nanoparticles for the immunization against reddish body iridovirus in turbots (Scophthalmus maximus). *Aquaculture* **2016**, *452*, 263–271, doi:10.1016/j.aquaculture.2015.11.013.

163. Zhou, L.; Yang, B.; Xu, L.; Jin, H.; Ge, X.; Guo, X.; Han, J.; Yang, H. Efficacy evaluation of three modified-live virus vaccines against a strain of porcine reproductive and respiratory syndrome virus NADC30-like. *Veterinary Microbiology* **2017**, *207*, 108–116, doi:10.1016/j.vetmic.2017.05.031.

164. Zhou, Y.S.; Zhang, Y.; Wang, H.N.; Fan, W.Q.; Yang, X.; Zhang, A.Y.; Zeng, F.Y.; Zhang, Z.K.; Cao, H.P.; Zeng, C. Establishment of reverse genetics system for infectious bronchitis virus attenuated vaccine strain H120. *Veterinary Microbiology* **2013**, *162*, 53–61, doi:10.1016/j.vetmic.2012.08.013.

165. Zhu, B.; Liu, G.-L.; Gong, Y.-X.; Ling, F.; Wang, G.-X. Protective immunity of grass carp immunized with DNA vaccine encoding the vp7 gene of grass carp reovirus using carbon nanotubes as a carrier molecule. *Fish & Shellfish Immunology* **2015**, *42*, 325–334, doi:10.1016/j.fsi.2014.11.026.

166. ZHU, Y.; ZOU, X.; BAO, H.; SUN, P.; MA, X.; LIU, Z.; FAN, H.; ZHAO, Q. Insertion site of FLAG on foot-and-mouth disease virus VP1 G-H loop affects immunogenicity of FLAG. *Journal of Integrative Agriculture* **2018**, *17*, 1655–1666, doi:10.1016/S2095-3119(18)61916-1.

167. CHENG, C.; YANG, J.; YAN, H.; BEI, X.; ZHANG, Y.; LU, Z.; ZHONG, G. Expressing p20 hairpin RNA of Citrus tristeza virus confers Citrus aurantium with tolerance/resistance against stem pitting and seedling yellow CTV strains. *Journal of Integrative Agriculture* **2015**, *14*, 1767–1777, doi:10.1016/S2095-3119(14)60937-0.

168. El-Mohtar, C.; Dawson, W.O. Exploring the limits of vector construction based on Citrus tristeza virus. *Virology* **2014**, *448*, 274–283, doi:10.1016/j.virol.2013.10.017.

169. Huang, W.; Reyes-Caldas, P.; Mann, M.; Seifbarghi, S.; Kahn, A.; Almeida, R.P.; Béven, L.; Heck, M.; Hogenhout, S.A.; Coaker, G. Bacterial Vector-Borne Plant Diseases: Unanswered Questions and Future Directions. *Molecular Plant* **2020**, *13*, 1379–1393, doi:10.1016/j.molp.2020.08.010.

170. Killiny, N.; Hijaz, F.; Harper, S.J.; Dawson, W.O. Effects of Citrus tristeza closterovirus infection on phloem sap and released volatile organic compounds in Citrus macrophylla. *Physiological and Molecular Plant Pathology* **2017**, *98*, 25–36, doi:10.1016/j.pmpp.2017.03.003.

171. Killiny, N.; Nehela, Y.; George, J.; Rashidi, M.; Stelinski, L.L.; Lapointe, S.L. Phytoene desaturase-silenced citrus as a trap crop with multiple cues to attract Diaphorina citri, the vector of Huanglongbing. *Plant Science* **2021**, *308*, 110930, doi:10.1016/j.plantsci.2021.110930.

172. Liu, C.; Liu, H.; Hurst, J.; Timko, M.P.; Zhou, C. Recent Advances on Citrus yellow vein clearing virus in Citrus. *Horticultural Plant Journal* **2020**, *6*, 216–222, doi:10.1016/j.hpj.2020.05.001.

173. Liu, Y.; Wang, G.; Wang, Z.; Yang, F.; Wu, G.; HONG, N. Identification of differentially expressed genes in response to infection of a mild Citrus tristeza virus isolate in Citrus aurantifolia by suppression subtractive hybridization. *Scientia Horticulturae* **2012**, *134*, 144–149, doi:10.1016/j.scienta.2011.11.022.

174. LIU, Z.; SUNZHU, Y.; ZHOU, X.; HONG, J.; WU, J. Monoclonal antibody-based serological detection of Citrus yellow vein clearing virus in citrus groves. *Journal of Integrative Agriculture* **2017**, *16*, 884–891, doi:10.1016/S2095-3119(16)61475-2.

175. Salem, R.; Arif, I.A.; Salama, M.; Osman, G.E. Polyclonal antibodies against the recombinantly expressed coat protein of the Citrus psorosis virus. *Saudi Journal of Biological Sciences* **2018**, *25*, 733–738, doi:10.1016/j.sjbs.2017.10.018.

176. Ali, G.; Abma-Henkens, M.H.; van der Werf, W.; Hemerik, L.; Vlak, J.M. Genotype assembly, biological activity and adaptation of spatially separated isolates of Spodoptera litura nucleopolyhedrovirus. *Journal of Invertebrate Pathology* **2018**, *153*, 20–29, doi:10.1016/j.jip.2018.01.009.

177. Beperet, I.; Simón, O.; Williams, T.; López-Ferber, M.; Caballero, P. The "11K" gene family members sf68, sf95 and sf138 modulate transmissibility and insecticidal properties of Spodoptera frugiperda multiple nucleopolyhedrovirus. *Journal of Invertebrate Pathology* **2015**, *127*, 101–109, doi:10.1016/j.jip.2015.03.008.

178. Bernal, A.; Williams, T.; Hernández-Suárez, E.; Carnero, A.; Caballero, P.; Simón, O. A native variant of Chrysodeixis chalcites nucleopolyhedrovirus: The basis for a promising bioinsecticide for control of C. chalcites on Canary Islands' banana crops. *Biological Control* **2013**, *67*, 101–110, doi:10.1016/j.biocontrol.2013.08.006.

179. Bixby, A.J.; Potter, D.A. Influence of endophyte (Neotyphodium lolii) infection of perennial ryegrass on susceptibility of the black cutworm (Lepidoptera: Noctuidae) to a baculovirus. *Biological Control* **2010**, *54*, 141–146, doi:10.1016/j.biocontrol.2010.04.003.

180. Bouneb, M.; Turchetti, T.; Nannelli, R.; Roversi, P.F.; Paoli, F.; Danti, R.; Simoni, S. Occurrence and transmission of mycovirus Cryphonectria hypovirus 1 from dejecta of Thyreophagus corticalis (Acari, Acaridae). *Fungal Biology* **2016**, *120*, 351–357, doi:10.1016/j.funbio.2015.11.004.

181. Cabodevilla, O.; Ibañez, I.; Simón, O.; Murillo, R.; Caballero, P.; Williams, T. Occlusion body pathogenicity, virulence and productivity traits vary with transmission strategy in a nucleopolyhedrovirus. *Biological Control* **2011**, *56*, 184–192, doi:10.1016/j.biocontrol.2010.10.007.

182. Carpio, C.; Dangles, O.; Dupas, S.; Léry, X.; López-Ferber, M.; Orbe, K.; Páez, D.; Rebaudo, F.; Santillán, A.; Yangari, B.; et al. Development of a viral biopesticide for the control of the Guatemala potato tuber moth Tecia solanivora. *Journal of Invertebrate Pathology* **2013**, *112*, 184–191, doi:10.1016/j.jip.2012.11.014.

183. Castro, M.E.B.; Melo, F.L.; Tagliari, M.; Inglis, P.W.; Craveiro, S.R.; Ribeiro, Z.M.A.; Ribeiro, B.M.; Báo, S.N. The genome sequence of Condylorrhiza vestigialis NPV, a novel baculovirus for the control of the Alamo moth on Populus spp. in Brazil. *Journal of Invertebrate Pathology* **2017**, *148*, 152–161, doi:10.1016/j.jip.2017.06.013.

184. Chen, Y.-Y.; Lin, J.-W.; Fan, Y.-C.; Tu, W.-C.; Chang, G.-J.J.; Chiou, S.-S. First detection of the Africa/Caribbean/Latin American subtype of Culex flavivirus in Asian country, Taiwan. *Comparative Immunology, Microbiology and Infectious Diseases* **2013**, *36*, 387–396, doi:10.1016/j.cimid.2013.02.001.

185. CHEN, Z.; YANG, Y.; ZHANG, J.; JIN, M.; XIAO, Y.; XIA, Z.; LIU, Y.; YU, S.; YANG, Y.; WANG, Y.; et al. Susceptibility and tissue specificity of Spodoptera frugiperda to

Junonia coenia densovirus. *Journal of Integrative Agriculture* **2021**, *20*, 840–849, doi:10.1016/S2095-3119(20)63163-X.

186. Del-Angel, C.; Lasa, R.; Mercado, G.; Rodríguez-del-Bosque, L.A.; Caballero, P.; Williams, T. Acquisition of lethal infection, hypermobility and modified climbing behavior in nucleopolyhedrovirus infected larvae of Anticarsia gemmatalis. *Biological Control* **2018**, *125*, 90–97, doi:10.1016/j.biocontrol.2018.07.006.
187. Fister, S.; Fuchs, S.; Stessl, B.; Schoder, D.; Wagner, M.; Rossmanith, P. Screening and characterisation of bacteriophage P100 insensitive Listeria monocytogenes isolates in Austrian dairy plants. *Food Control* **2016**, *59*, 108–117, doi:10.1016/j.foodcont.2015.05.026.
188. García-Banderas, D.; Tamayo-Mejía, F.; Pineda, S.; La Rosa, J.I.F. de; Lasa, R.; Chavarrieta-Yáñez, J.M.; Gervasio-Rosas, E.; Zamora-Avilés, N.; Morales, S.I.; Ramos-Ortiz, S.; et al. Biological characterization of two Spodoptera frugiperda nucleopolyhedrovirus isolates from Mexico and evaluation of one isolate in a small-scale field trial. *Biological Control* **2020**, *149*, 104316, doi:10.1016/j.biocontrol.2020.104316.
189. Gasque, S.N.; van Oers, M.M.; Ros, V.I.D. Where the baculoviruses lead, the caterpillars follow: baculovirus-induced alterations in caterpillar behaviour. *Current Opinion in Insect Science* **2019**, *33*, 30–36, doi:10.1016/j.cois.2019.02.008.
190. Gupta, R.K.; Gani, M.; Kaul, V.; Bhagat, R.M.; Bali, K.; Samnotra, R.K. Field evaluation of Lymantria obfuscata multiple nucleopolyhedrovirus for the management of Indian gypsy moth in Jammu & Kashmir, India. *Crop Protection* **2016**, *80*, 149–158, doi:10.1016/j.cropro.2015.11.008.
191. Infante-Rodríguez, D.A.; Berber, J.J.; Mercado, G.; Valenzuela-González, J.; Muñoz, D.; Williams, T. Earthworm mediated dispersal of baculovirus occlusion bodies: Experimental evidence from a model system. *Biological Control* **2016**, *100*, 18–24, doi:10.1016/j.biocontrol.2016.05.005.
192. Jayachandran, B.; Hussain, M.; Asgari, S. Regulation of Helicoverpa armigera ecdysone receptor by miR-14 and its potential link to baculovirus infection. *Journal of Invertebrate Pathology* **2013**, *114*, 151–157, doi:10.1016/j.jip.2013.07.004.
193. Kariithi, H.M.; Meki, I.K.; Boucias, D.G.; Abd-Alla, A.M.M. Hytrosaviruses: current status and perspective. *Current Opinion in Insect Science* **2017**, *22*, 71–78, doi:10.1016/j.cois.2017.05.009.
194. Kariithi, H.M.; van Lent, J.; van Oers, M.M.; Abd-Alla, A.M.; Vlak, J.M. Proteomic footprints of a member of Glossinavirus (Hytrosaviridae): An expeditious approach to virus control strategies in tsetse factories. *Journal of Invertebrate Pathology* **2013**, *112*, S26-S31, doi:10.1016/j.jip.2012.07.016.
195. Kemp, E.M.; Woodward, D.T.; Cory, J.S. Detection of single and mixed covert baculovirus infections in eastern spruce budworm, Choristoneura fumiferana populations. *Journal of Invertebrate Pathology* **2011**, *107*, 202–205, doi:10.1016/j.jip.2011.05.015.
196. Lasa, R.; Guerrero-Analco, J.A.; Monribot-Villanueva, J.L.; Mercado, G.; Williams, T. Why do Spodoptera exigua multiple nucleopolyhedrovirus occlusion bodies lose insecticidal activity on amaranth (Amaranthus hypocondriacus L.)? *Biological Control* **2018**, *126*, 74–82, doi:10.1016/j.biocontrol.2018.07.014.
197. Lietze, V.-U.; Keesling, J.E.; Lee, J.A.; Vallejo, C.R.; Geden, C.J.; Boucias, D.G. Muscavirus (MdSGHV) disease dynamics in house fly populations – How is this virus transmitted and has it potential as a biological control agent? *Journal of Invertebrate Pathology* **2013**, *112*, S40-S43, doi:10.1016/j.jip.2012.07.017.
198. Llopis-Giménez, A.; Maria González, R.; Millán-Leiva, A.; Catalá, M.; Llacer, E.; Urbaneja, A.; Herrero, S. Novel RNA viruses producing simultaneous covert infections in

Ceratitis capitata. Correlations between viral titers and host fitness, and implications for SIT programs. *Journal of Invertebrate Pathology* **2017**, *143*, 50–60, doi:10.1016/j.jip.2016.11.014.

199. Nalcacioglu, R.; Muratoglu, H.; Yesilyurt, A.; van Oers, M.M.; Vlak, J.M.; Demirbag, Z. Enhanced insecticidal activity of Chilo iridescent virus expressing an insect specific neurotoxin. *Journal of Invertebrate Pathology* **2016**, *138*, 104–111, doi:10.1016/j.jip.2016.06.010.
200. Ottati, S.; Persico, A.; Rossi, M.; Bosco, D.; Vallino, M.; Abbà, S.; Molinatto, G.; Palmano, S.; Balestrini, R.; Galetto, L.; et al. Biological characterization of Euscelidius variegatus iflavirus 1. *Journal of Invertebrate Pathology* **2020**, *173*, 107370, doi:10.1016/j.jip.2020.107370.
201. Parreño, R.; Torres, S.; Almagro, L.; Belló-Pérez, M.; Estepa, A.; Perez, L. Induction of viral interference by IPNV-carrier cells on target cells: A cell co-culture study. *Fish & Shellfish Immunology* **2016**, *58*, 483–489, doi:10.1016/j.fsi.2016.09.052.
202. Rabalski, L.; Krejmer-Rabalska, M.; Skrzecz, I.; Wasag, B.; Szewczyk, B. An alphabaculovirus isolated from dead Lymantria dispar larvae shows high genetic similarity to baculovirus previously isolated from Lymantria monacha – An example of adaptation to a new host. *Journal of Invertebrate Pathology* **2016**, *139*, 56–66, doi:10.1016/j.jip.2016.07.011.
203. Saunders, G.; Cooke, B.; McColl, K.; Shine, R.; Peacock, T. Modern approaches for the biological control of vertebrate pests: An Australian perspective. *Biological Control* **2010**, *52*, 288–295, doi:10.1016/j.biocontrol.2009.06.014.
204. Serrano, A.; Pijlman, G.P.; Vlak, J.M.; Muñoz, D.; Williams, T.; Caballero, P. Identification of Spodoptera exigua nucleopolyhedrovirus genes involved in pathogenicity and virulence. *Journal of Invertebrate Pathology* **2015**, *126*, 43–50, doi:10.1016/j.jip.2015.01.008.
205. Shikano, I.; McCarthy, E.M.; Elderd, B.D.; Hoover, K. Plant genotype and induced defenses affect the productivity of an insect-killing obligate viral pathogen. *Journal of Invertebrate Pathology* **2017**, *148*, 34–42, doi:10.1016/j.jip.2017.05.001.
206. Simón, O.; Palma, L.; Williams, T.; López-Ferber, M.; Caballero, P. Analysis of a naturally-occurring deletion mutant of Spodoptera frugiperda multiple nucleopolyhedrovirus reveals sf58 as a new per os infectivity factor of lepidopteran-infecting baculoviruses. *Journal of Invertebrate Pathology* **2012**, *109*, 117–126, doi:10.1016/j.jip.2011.10.010.
207. Souza, M.L.; Sanches, M.M.; Souza, D.A. de; Faria, M.; Espinel-Correal, C.; Sihler, W.; Lopes, R.B. Within-host interactions of Metarhizium rileyi strains and nucleopolyhedroviruses in Spodoptera frugiperda and Anticarsia gemmatalis (Lepidoptera: Noctuidae). *Journal of Invertebrate Pathology* **2019**, *162*, 10–18, doi:10.1016/j.jip.2019.01.006.
208. Takatsuka, J. Lymantria mathura nucleopolyhedrovirus: Identification, occurrence and genetic diversity in Iwate Prefecture, Japan. *Journal of Invertebrate Pathology* **2016**, *138*, 1–9, doi:10.1016/j.jip.2016.05.006.
209. Takatsuka, J.; Okuno, S.; Ishii, T.; Nakai, M.; Kunimi, Y. Fitness-related traits of entomopoxviruses isolated from Adoxophyes honmai (Lepidoptera: Tortricidae) at three localities in Japan. *Journal of Invertebrate Pathology* **2010**, *105*, 121–131, doi:10.1016/j.jip.2010.04.010.

210. Tian, Z.; Zha, M.; Cai, L.; Michaud, J.P.; Cheng, J.; Shen, Z.; Liu, X.; Liu, X. FoxO-promoted peroxiredoxin1 expression induced by Helicoverpa armigera single nucleopolyhedrovirus infection mediates host development and defensive responses. *Ecotoxicology and Environmental Safety* **2022**, *234*, 113414, doi:10.1016/j.ecoenv.2022.113414.

211. Yang, Q.; Ding, Y.; Nie, R.; Yao, L.; Wang, X.; Zhou, M.; Wang, J.; Wang, X. Characterization of a novel T7-like Salmonella Typhimurium (ATCC13311) bacteriophage LPST144 and its endolysin. *LWT* **2020**, *123*, 109034, doi:10.1016/j.lwt.2020.109034.

212. Zwart, M.P.; van der Werf, W.; Georgievska, L.; van Oers, M.M.; Vlak, J.M.; Cory, J.S. Mixed-genotype infections of Trichoplusia ni larvae with Autographa californica multicapsid nucleopolyhedrovirus: Speed of action and persistence of a recombinant in serial passage. *Biological Control* **2010**, *52*, 77–83, doi:10.1016/j.biocontrol.2009.10.002.
